# Implications for Cation Selectivity and Evolution by a Novel Cation Diffusion Facilitator Family Member From the Moderate Halophile *Planococcus dechangensis*

**DOI:** 10.3389/fmicb.2019.00607

**Published:** 2019-03-22

**Authors:** Tong Xu, Huiwen Chen, Jincheng Li, Shan Hong, Li Shao, Xiutao Zheng, Qiao Zou, Yuting Wang, Sijia Guo, Juquan Jiang

**Affiliations:** Department of Microbiology and Biotechnology, College of Life Sciences, Northeast Agricultural University, Harbin, China

**Keywords:** cation selectivity, cation diffusion facilitator family, Na^+^(Li^+^, K^+^)/H^+^ antiporter, facilitated Zn^2+^ diffusion, secondary transporter, moderate halophile

## Abstract

In the cation diffusion facilitator (CDF) family, the transported substrates are confined to divalent metal ions, such as Zn^2+^, Fe^2+^, and Mn^2+^. However, this study identifies a novel CDF member designated MceT from the moderate halophile *Planococcus dechangensis*. MceT functions as a Na^+^(Li^+^, K^+^)/H^+^ antiporter, together with its capability of facilitated Zn^2+^ diffusion into cells, which have not been reported in any identified CDF transporters as yet. MceT is proposed to represent a novel CDF group, Na-CDF, which shares significantly distant phylogenetic relationship with three known CDF groups including Mn-CDF, Fe/Zn-CDF, and Zn-CDF. Variation of key function-related residues to “Y44-S48-Q150” in two structural motifs explains a significant discrimination in cation selectivity between Na-CDF group and three major known CDF groups. Functional analysis via site-directed mutagenesis confirms that MceT employs Q150, S158, and D184 for the function of MceT as a Na^+^(Li^+^, K^+^)/H^+^ antiporter, and retains D41, D154, and D184 for its facilitated Zn^2+^ diffusion into cells. These presented findings imply that MceT has evolved from its native CDF family function to a Na^+^/H^+^ antiporter in an evolutionary strategy of the substitution of key conserved residues to “Q150-S158-D184” motif. More importantly, the discovery of MceT contributes to a typical transporter model of CDF family with the unique structural motifs, which will be utilized to explore the cation-selective mechanisms of secondary transporters.

## Introduction

Cation diffusion facilitator (CDF) family proteins are ubiquitous secondary transmembrane transporters in all three kingdoms of living organisms including bacteria, archaea and eukaryotes, which play an important role in the homeostasis of divalent metal cations (Me^2+^) including Zn^2+^, Cd^2+^, Co^2+^, Fe^2+^, Ni^2+^, Mn^2+^ and possibly Cu^2+^ and Pb^2+^ (Paulsen and Saier, 1997; Haney et al., 2005; Montanini et al., 2007). Most members of this family function as Me^2+^/H^+^ antiporters, which utilize the proton motive force for the transport of divalent metal ions from the cytoplasm to the outside of cells or into subcellular compartments. In addition, *Escherichia coli* ZitB and *Bacillus subtilis* CzcD can exchange Me^2+^ for H^+^ and also K^+^ (Guffanti et al., 2002; Lee et al., 2002). Based on the phylogenetic analysis, CDF transporters are classified into three major groups with different metal ion specificity: (i) Mn-CDF with the sole substrate of Mn^2+^; (ii) Fe/Zn CDF with the substrates of Fe^2+^ and Zn^2+^ and also other metal ions; (iii) Zn-CDF with substrates of Zn^2+^ and other metal ions but not including Fe^2+^ or Mn^2+^ (Montanini et al., 2007). Most CDF members possess a cation-transporting transmembrane domain (TMD) composed of six transmembrane helices (TMHs), followed by a ∼100 aa long cytoplasmic regulatory C-terminal domain (CTD) folded into two α helices and three β strands (Paulsen and Saier, 1997; Kolaj-Robin et al., 2015).

X-ray structure of *E. coli* YiiP in complex with Zn^2+^ provides an archetypal 3D model for CDF members (Lu and Fu, 2007; Lu et al., 2009). Of three Zn^2+^-binding sites (A-C) of YiiP, the major binding site A is located in the center of TMD and consists of four coordinating residues: D45 and D49 (DD) in TMH2 and H153 and D157 (HD) in TMH5, whereas other two binding sites (B and C) are located in the cytoplasmic Loop 2,3 and CTD domain, respectively. Six TMHs are grouped into two bundles with four (TMH1-TMH2-TMH4-TMH5) and two (TMH3-TMH6) helices (Coudray et al., 2013). So far, DD, ND, or HD motifs in TMH2 and HD motif in TMH5 have been widely accepted to be responsible for ionic selectivity between divalent metal ions (Montanini et al., 2007; Kolaj-Robin et al., 2015; Martin and Giedroc, 2016). For example, residue swapping of HD to DD of human ZnT5 or ZnT8 abolished their ionic selectivity against Cd^2+^, but had no effect on Zn^2+^ transport (Hoch et al., 2012). Mutation of DD to HD in TMH2 resulted in the Zn^2+^and Cd^2+^ specificity of *E. coli* YiiP to its preferred Zn^2+^ (Hoch et al., 2012). An H90D mutation in TMH2 of rice CDF OsMTP1 abolished Zn^2+^ transport but improved Fe^2+^ transport (Menguer et al., 2013). Mutation of HD to ND in TMH2 of human ZnT1 resulted in the loss of its native Zn^2+^ transport activity and the conversion into a Mn^2+^ efflux transporter, as human ZnT10 with ND motif in its TMH2 (Nishito et al., 2016).

Na^+^/H^+^ antiporters are a category of secondary transmembrane transporters that catalyze the exchange of Na^+^ for H^+^, which play a major role in maintaining intracellular pH and Na^+^ homeostasis (Padan and Landau, 2016). Under high saline-alkaline stress, halophiles should have been driven to evolve a larger number of Na^+^/H^+^ antiporters to stabilize their intracellular osmotic and ionic state. This has been strongly supported by our recent reports on several novel Na^+^/H^+^ antiporters such as UPF0118, UmpAB, RDD, and MdrP from the slight or moderate halophiles (Dong et al., 2017; Meng et al., 2017; Abdel-Motaal et al., 2018; Shao et al., 2018). Secondary transporters are proposed to share similar structures but different transported substrates (Shi, 2013; Yan, 2013), and thus a transporter model may alter the substrate selectivity by changing conserved functional residues. Therefore, some proteins from halophiles may have evolved from its native family functions to Na^+^ efflux transporters by changing conserved functional residues, but remaining the homologies and similar structures.

In this study, a moderate halophile, *Planococcus dechangensis* NEAU-ST10-9^T^ (Wang et al., 2015) was used as a research object for gene mining, in order to screen Na^+^/H^+^ antiporters especially novel ones, and even to discover new functions or structure models of members within known ionic transporter families. Consequently, a novel CDF transporter, MceT, was obtained and identified to function as a Na^+^(Li^+^, K^+^)/H^+^ antiporter, together with the capability of facilitated Zn^2+^ diffusion into cells, which has not been reported in identified CDF members as yet. The function-related structural motifs for Na^+^ efflux were identified through site-directed mutagenesis of conserved residues. These presented findings imply that MceT has evolved from a Zn^2+^-efflux model of CDF members to a novel Na^+^-efflux model of Na^+^/H^+^ antiporters through the substitution of key conserved residues in its structural motifs.

## Materials and Methods

### Bacterial Strains, Plasmids, and Growth Conditions

Strains and plasmids are presented in Supplementary Table 1. *P. dechangensis* NEAU-ST10-9^T^ was grown in a modified S-G liquid medium as previously described (Wang et al., 2015). A Na^+^/H^+^ antiporter-deficient mutant *E*. *coli* KNabc (Δ*nhaA*Δ*nhaB*Δ*chaA*) was used for NaCl complementation, growth tests and Na^+^/H^+^ antiport activity assays. A Zn^2+^-sensitive *E*. *coli* mutant KZAB04 (Δ*zntA*Δ*zitB*) was used for ZnCl_2_ resistance experiments and intracellular Zn^2+^ accumulation analysis. This mutant was constructed with *E*. *coli* DH5α as an original strain by inserting a kanamycin resistance gene from pBBR1MCS-2 into *zntA* and a gentamicin resistance gene from pBBR1MCS-5 into *zitB*, via homologous recombination with the aid of pKD46 containing a λ Red recombinase system (Datsenko and Wanner, 2000). *E*. *coli* strains were cultured to OD_600__nm_ at 1.0 in Luria-Bertani (LB) broth (1% tryptone, 0.5% yeast, 1% NaCl), LBO broth (LB without the addition of NaCl) or LBK broth (LB with the addition of 86 mM KCl instead of NaCl), followed by the inoculation into the same fresh media and incubation at 37°C within 24 h. In the physiological experiments, NaCl (0–0.4 M), LiCl (0–10 mM), or ZnCl_2_ (0–0.75 mM) was added into the indicated media. pH was adjusted by the addition of 10 mM Tris-HCl buffer (7.0–8.5) at the final concentration to the tested media supplemented by 50 mM NaCl, which is essential for the alkaline pH resistance of Na^+^/H^+^ antiporters as previously described (Krulwich et al., 2011; Quinn et al., 2012; Meng et al., 2014; Padan, 2014).

### Isolation and Subcloning of a Na^+^/H^+^ Antiporter Gene Candidate

The pUC18 vector, which was digested by *Bam*HI and dephosphorylated by a bacterial alkaline phosphatase, was ligated with *Sau*3AI-partially-digested genomic DNA fragments from strain NEAU-ST10-9^T^ as described in our previous study (Meng et al., 2017). After electroporation into *E. coli* KNabc cells, the recombinant plasmid designated pUC-S5 was separated by functional complementation with *E. coli* KNabc on LBK medium plates containing 0.2 M NaCl. The 2712 bp nucleotide sequence was submitted to the GenBank database with the accession No. MH845411. For the subcloning of *mceT* gene, expression vector pTrcHisB-mceT was constructed through the fusion of the ORF sequence of *mceT* gene in frame with an N-terminal 6xHis tag followed by an enterokinase cleavage site into an expression vector, pTrcHisB. The gene *czcD* encoding an identified CDF member from *B. subtilis* subsp. *subtilis* strain 168 (Guffanti et al., 2002), was cloned as the positive control of a Zn^2+^ efflux transporter via the same cloning strategy as *mceT*. The resultant constructs, pTrcHisB-mceT and pTrcHisB-czcD were verified by sequencing. Primers are listed in Supplementary Table 2.

### Preparation of Everted Membrane Vesicles

Everted membrane vesicles were prepared as previously described (Rosen and Tsuchiya, 1979) with a minorly-modified buffer containing 10 mM Tris-HCl (pH7.5), 140 mM choline chloride, 0.5 mM dithiothreitol (DTT), 1 mM phenylmethanesulfonyl fluoride (PMSF) and 250 mM sucrose. Cells of *E. coli* KNabc carrying pTrcHisB-mceT or its variants, as well as the empty vector pTrcHisB, were harvested and everted by one passage through a JG-1A French Press (NingBo Scientz Biotechnology Co., Ltd., China) at 1000 psi system pressure, and cell debris was removed by centrifugation at 12,000 g for 15 min. The resultant membrane vesicles were collected by centrifugation at 100,000 g for 60 min. The above procedures were performed at 4 °C. The pellets were resuspended in the same buffer, and stored at −80°C. During this course, cell extract and cytoplasmic fraction membrane fraction existing as everted membrane vesicles were sampled, respectively, for the determination of expression and localization of MceT or its variants, as described in our recent studies (Abdel-Motaal et al., 2018; Shao et al., 2018).

### Western Blot

Protein samples with 30 μg for each were separated on 12% SDS-polyacrylamide gels and blotted (Bio-Rad Laboratories, Inc., China) onto polyvinylidene difluoride membranes. Western blot detection was performed by using a polyclonal mouse anti-6xHis tag antibody (Beyotime Biotechnology Co. Ltd., Shanghai, China) and an HRP-labeled goat anti-mouse IgG(H+L) (Nachuan Biotechnology Co., Ltd., Changchun, China). The blots were visualized using a BeyoECL Star kit (Beyotime Biotechnology Co. Ltd., Shanghai, China) and recorded by a Tanon-5200 imaging system (Tanon Co. Ltd., China).

### Measurements of Na^+^(Li^+^, K^+^)/H^+^ Antiport Activity by Fluorescence

Na^+^(Li^+^, K^+^)/H^+^ antiport activity assays were performed as previously described (Bassilana et al., 1984; Nakamura et al., 1986; Goldberg et al., 1987). Everted vesicles containing approximately 100 μg of total membrane protein were added into a 2 ml reaction buffer containing 140 mM choline chloride, 250 mM sucrose, 1 μM acridine orange and 10 mM BTP/HCl adjusted to the indicated pH. Respiration-dependent formation of ΔpH was initiated by the addition of 10 mM Tris-D-lactate, which resulted in the quenching of acridine orange fluorescence. Antiport activity was estimated from the dequenching percentage after the addition of NaCl, LiCl or KCl at the final concentration of 5 mM. Fluorescence was measured with a fluorescence spectrophotometer F-7000 (Hitachi High-Technologies, Japan) with excitation at 490 nm and emission at 530 nm. The apparent affinity of the antiporter for the cations was estimated through the calculation of K_0.5_ values, which were obtained by fitting the antiport activity as the functions of corresponding cation concentrations followed by non-linear regression analysis using the software Prism 7.0.

### Analysis of Intracellular Zn^2+^ Accumulation

Overnight-grown cultures were diluted 100-fold into 200 ml of fresh LBK broth and continued to grow till OD_600nm_ reached 1.0. Cells were harvested and washed two times with a buffer containing 140 mM choline chloride, 0.2% glucose, 10 mM Tris-HCl at pH 7.5 and then resuspended and adjusted to the same total protein concentration in 2 ml of the same buffer at 4°C. Four moicroliter of cell suspensions were transferred into 96 μl of the same buffer and incubated at 25°C for 10 min before the reaction. In order to start the reaction, appropriate amounts of ZnCl_2_ were added to the indicated final concentrations. After incubation at 25°C, the reaction was terminated by the addition of 4 ml of the same ice-pre-cooled buffer and immediately filtered through a polyethersulfone (PES) membrane (0.45 μm). Ten microliter of the same ice-pre-cooled buffer was passed through the PES membrane to wash the cells. Finally, 2.5 ml of 5% trichloroacetic acid (TCA) was passed five times through the PES membrane to lyse the cells and dissolve the intracellular Zn^2+^. Zn^2+^ contents were determined using an atomic absorption spectrophotometer AA-6650 (Shimadzu, Kyoto, Japan).

### Site-Directed Mutagenesis

Site-directed mutagenesis was performed with a Fast Mutagenesis System Kit (Transgen Biotech, Beijing, China), according to the manufacturer’s instructions. Variants were generated by PCR using pTrcHisB-mceT as a template and mutagenic primers (Supplementary Table 2), and verified for the sequence accuracy by sequencing.

### Protein Concentration

Protein concentration was determined by the Bradford protein assay with bovine serum albumin as the standard (Bradford, 1976).

### Bioinformatic Analysis

DNA sequencing was carried out by Beijing Genomics Institute (Beijing, China). ORFs were deduced by DNAMAN 8.0 and aligned using BlastP at the NCBI website https://blast.ncbi.nlm.nih.gov/Blast.cgi. Promoters were predicted at the website http://www.fruitfly.org/seq_tools/promoter.html (Reese, 2001). Topological analysis was performed at the PredictProtein website https://www.predictprotein.org. CDF members were downloaded from the TCDB database (Saier et al., 2016) at the website http://www.tcdb.org. Amino acid sequence logos were created by submitting the multiple sequence alignment to the WebLogo 3 website http://weblogo.threeplusone.com/. The taxonomy of the hosts was recognized in the UniProtKB/Swiss-Prot database at the website https://www.uniprot.org/taxonomy/. Protein sequences were aligned with the software ClustalX 1.83, followed by the construction of a neighbor-joining phylogenetic tree via the software MEGA 5.0 on the basis of a bootstrap analysis on the clustering stability (1000 replications) (Saitou and Nei, 1986). The modeled structure of MceT was constructed using the Swiss-Model server at the website https://www.swissmodel.expasy.org/interactive. Structure assessment enclosed in Swiss-Model server was used to verify the reliability of the modeled structure of MceT.

## Results

### Screening for a Na^+^/H^+^ Antiporter Gene Candidate

In this study, a Na^+^/H^+^ antiporter-deficient *E. coli* mutant KNabc (Δ*nhaA*, Δ*nhaB*, Δ*chaA*) (Nozaki et al., 1996) was employed to screen Na^+^/H^+^ antiporter gene from strain NEAU-ST10-9^T^ by functional complementation on LBK medium plates containing 0.2 M NaCl, which is the upper limit for the growth of *E. coli* KNabc and routinely selected as the growth condition for the screening of Na^+^/H^+^ antiporter genes. As a result, a recombinant plasmid designated pUC-S5 with a 2712 bp digestion fragment succeeded in complementing with *E*. *coli* KNabc. Sequence analysis showed three open reading frames (ORFs 1–3) in the fragment (Supplementary Figure 1). ORF1 shares the highest identity (48%) with a hypothetical protein (accession version No. AQU78343.1) from *Planococcus faecalis*, ORF2 shares the highest identity (69%) with a TetR/AcrR family transcriptional regulator (accession version WP_052144530.1) from *Bacillus okhensis*, and ORF3 shares the highest identity (63%) with a CDF transporter (accession version No. WP_084309370.1) from *Bacillus okhensis*. Each ORF is preceded by a predicted promoter and a Shine-Dalgarno (SD) sequence and also ORF3 is followed by one possible terminator (Supplementary Figure 1). It seems that each of them can be a Na^+^/H^+^ antiporter gene candidate.

However, each of three ORFs shares no identity with identified single-gene Na^+^/H^+^ antiporters or proteins reported to exhibit Na^+^/H^+^ antiport activity, the subunit of double-gene or multiple-gene Na^+^/H^+^ antiporters, and even predicted Na^+^/H^+^ antiporters. Topological analysis showed that ORF3 is the sole transmembrane protein consisting of six TMHs including TMH1 (6–33), TMH2 (35–62), TMH3 (74–104), TMH4 (113–135), TMH5 (142–173), and TMH6 (180–211), and two α helices including α1 (217–229) and α2 (264–281), and also three β stands including β1 (236–245), β2 (250–258) and β3 (288-295) (Supplementary Figure 2), which is a typical topological characteristic of CDF members (Lu and Fu, 2007; Coudray et al., 2013). Therefore, ORF3 may be the real Na^+^/H^+^ antiporter gene candidate. For the convenience of the following description, ORF3 was designated MceT on the basis of its main function as a monovalent cation efflux transporter.

### Alignment of MceT With Its Homologs

To confirm whether MceT belongs to CDF family, MceT was aligned with its putative homologs using BlastP at the NCBI website. Ten representative homologs were selected from the different species widely distributed in eight phyla (Supplementary Table 3) to show the multiple alignment with MceT (Supplementary Figure 2). In addition to the putative CDF member from *B. okhensis*, MceT shares 28–45% identities with the selected CDF members (Supplementary Table 3). Also, MceT and its homologs contain the signature sequence between TMH2 and TMH3, as is almost consistent with those of reported CDF members (Montanini et al., 2007). Moreover, MceT shares four relatively highly conserved motifs (Motifs 1–4) with its putative homologs (Supplementary Figure 2).

Because the above-mentioned homologs were predicted on the basis of sequence homology, all the identified and several putative CDF members were also downloaded from the TCDB database, and aligned with MceT using BlastP at the NCBI website. MceT shares ≤24% identities with eleven identified CDF members within the query cover range from 54 to 90% (Supplementary Table 3). These eleven proteins were also aligned with MceT to find out their homology and difference with MceT (Supplementary Figure 3). Interestingly, two motifs, Motif 2 and Motif 4, located in the beginning of TMH3 and TMH6 share almost the same sequence similarity whereas the other two motifs, Motif 1 and Motif 3, located in TMH2 and TMH5, respectively, are significantly varied at the corresponding positions to Y44, S48, and Q150 of MceT (Supplementary Figure 3), compared with those of identified CDF members (Montanini et al., 2007). Based on the above analysis, MceT may be different from identified CDF members, although it should belong to CDF family.

### Growth Tests for Salt Tolerance and Alkaline pH Resistance

**FIGURE 1:**
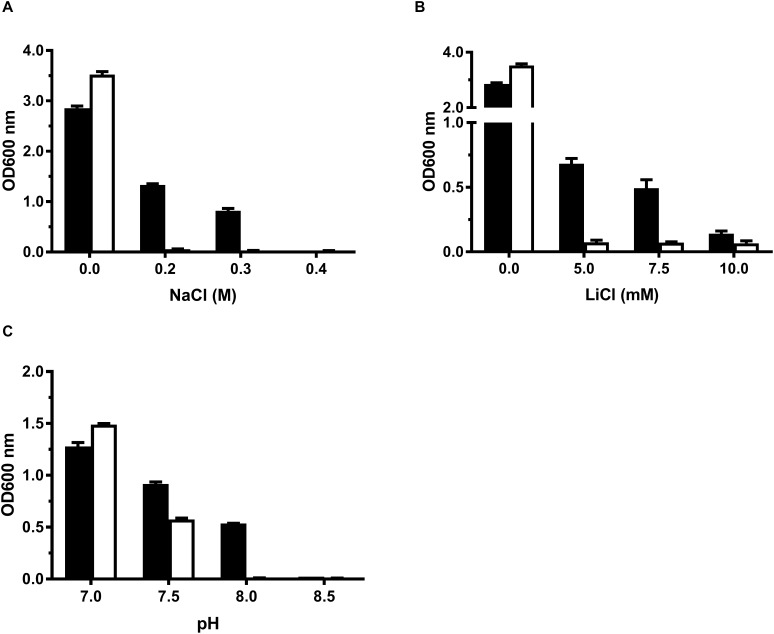
Growth tests for *E. coli* KNabc transformants under saline or alkaline conditions. For the growth tests under saline or alkaline conditions, *E. coli* KNabc/pTrcHisB-mceT (black column) and KNabc/pTrcHisB (white column) were grown in LBK broths containing 0–0.4 M NaCl **(A)** or 0–10 mM LiCl **(B),** or at the pH values from 7.0 to 8.5 supplemented by 50 mM NaCl **(C)**. The pre-cultures of *E. coli* KNabc transformant cells were grown to OD_600__nm_ at 1.0 in LBK broths at pH 7.0 at 37°C. The above-mentioned cell growth was ended after 24 h and the values for OD_600__nm_ then evaluated. Each data point represents Mean ± SD of three independent cultures.

For the functional analysis of MceT, the *mceT* gene was fused in frame with an N-terminal 6 × His tag of an expression vector pTrcHisB. As a preliminary test, the growth tests were carried out for salt tolerance and alkaline pH resistance using the resultant construct designated pTrcHisB-mceT and the empty vector pTrcHisB as a negative control (Supplementary Table 1). Expression of MceT in *E*. *coli* KNabc led to the increase of Na^+^ and Li^+^ tolerance of the host from 5 mM LiCl and 0.2 M NaCl to 0.3 M (Figure 1A) and 7.5 mM (Figure 1B), respectively. Also, expression of MceT significantly enhanced the host growth under alkaline pH conditions, especially at pH 8.0, in contrast to the empty vector (Figure 1C). Therefore, MceT is likely to function as a Na^+^/H^+^ antiporter.

### Establishment of MceT as a Transmembrane Protein by Western Blot

To establish the expression of MceT, cell extract, cytoplasmic fraction and membrane fraction were sampled during the preparation of everted membrane vesicles from *E. coli* KNabc carrying pTrcHisB-mceT and the empty vector pTrcHisB as a negative control, followed by the running in the SDS-PAGE (Supplementary Figure 4A) and detection by western blot (Supplementary Figure 4B). His tag-labeled MceT was detected in both cell extract and membrane fraction from *E. coli* KNabc/pTrcHisB-mceT, but not in those of KNabc/pTrcHisB (Supplementary Figure 4B). Also, no positive signal was detected in cytoplasmic fraction from *E. coli* KNabc/pTrcHisB-mceT or KNabc/pTrcHisB. These results reveal that MceT is indeed located in the cytoplasmic membrane of *E. coli* KNabc.

### Function of MceT as a Na^+^(Li^+^, K^+^)/H^+^ Antiporter

**FIGURE 2:**
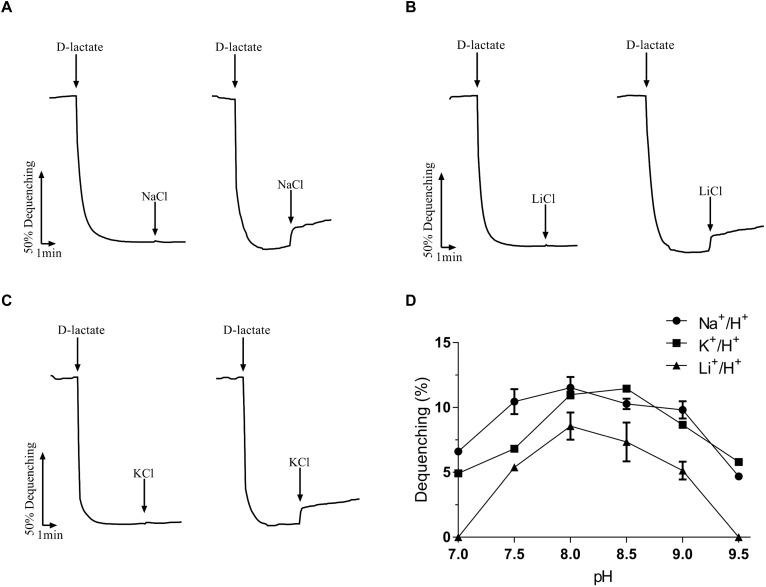
Na^+^/H^+^ antiporter activity measured by the acridine orange fluorescence quenching method. | Everted membrane vesicles (100 μg of total membrane protein) prepared from cells of *E. coli* KNabc/pTrcHisB-mceT (right) or KNabc/pTrcHisB (left) were added in a 2 ml reaction mixture. At the time points indicated, Tris-D-lactate was added at the final concentration of 10 mM to initiate fluorescence quenching due to the H^+^ transport caused by respiration. Then NaCl, LiCl or KCl was added at the final concentration of 5 mM to the assay mixture at the indicated time points. The transport activity at different pH values was determined by calculating the percentage of dequenching to total quenching (Dequenching %). The antiport activity at pH 8.0 for Na^+^/H^+^ **(A)**, Li^+^/H^+^ **(B)**, or at pH 8.5 for K^+^/H^+^ **(C)** was shown as the representatives. Also, the antiport activity pH profile **(D)** for Na^+^/H^+^ (filled circle), Li^+^/H^+^ (filled triangle) and K^+^/H^+^ (filled square) was also plotted at the indicated pH values. Each data point represents Mean ± SD of three independent experiments.

**FIGURE 3:**
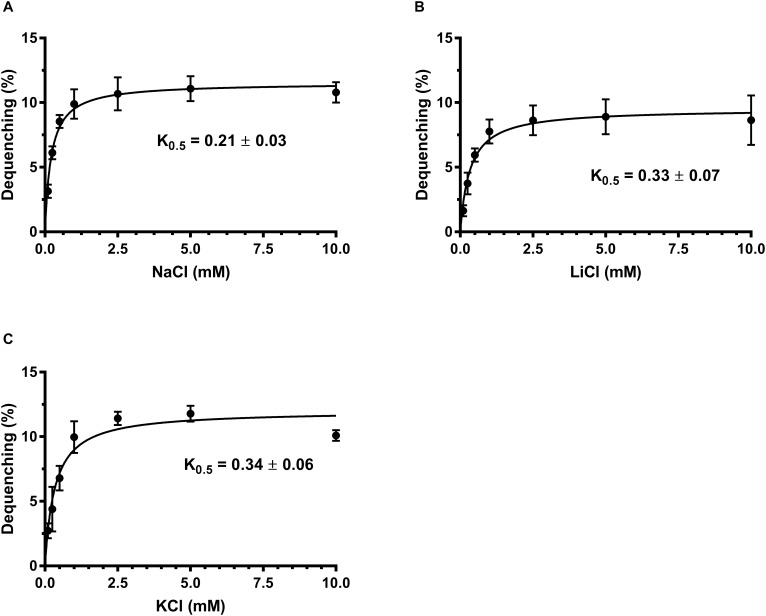
Calculation of K_0.5_ values of MceT for Na^+^, Li^+^, and K^+^. The antiport activity was determined at pH 8.0 for Na^+^/H^+^ **(A),** Li^+^/H^+^ **(B)**, and K^+^/H^+^ **(C)** at the cation concentrations varied from 0 to 10 mM. K_0.5_ values of MceT for the tested cations were calculated by fitting the antiport activity as the functions of the corresponding cation concentrations followed by non-linear regression analysis with the software Prism 7.0. Each data point represents Mean ± SD of three independent experiments.

Na^+^/H^+^ antiporters simultaneously possess Na^+^/H^+^ and Li^+^/H^+^ antiport activity (Krulwich et al., 2011; Padan, 2014), and also some of them exhibit K^+^/H^+^ antiport activity (Fujisawa et al., 2007; Meng et al., 2017; Abdel-Motaal et al., 2018; Shao et al., 2018). To identify the function of MceT, everted membrane vesicles were prepared from *E. coli* KNabc/pTrcHisB-mceT or KNabc/pTrcHisB and Na^+^ (Li^+^, K^+^)/H^+^ antiport activity was measured using a conventional fluorescence dequenching method with the acridine orange as a pH indicator. As a result, MceT exhibited Na^+^(Li^+^)/H^+^ (Figure 2A,B) antiport activity, and also K^+^/H^+^ (Figure 2C) antiport activity, which were detected within the pH range from 7.0 to 9.5 with the optimal for Na^+^(Li^+^)/H^+^ antiport activity at pH 8.0 and K^+^/H^+^ antiport activity at pH 8.0–8.5 (Figure 2D). K_0.5_ values of MceT were analyzed for Na^+^, K^+^, and Li^+^ in order to assess the apparent affinity of MceT for the cations, and calculated each of them to be 0.21 ± 0.03 mM (Figure 3A), 0.33 ± 0.07 mM (Figure 3B), and 0.34 ± 0.06 mM (Figure 3C), respectively. Therefore, MceT can function as a Na^+^(Li^+^, K^+^)/H^+^ antiporter.

### Zn^2+^ Sensitivity of *E. coli* KZAB04 Exacerbated by MceT

**FIGURE 4:**
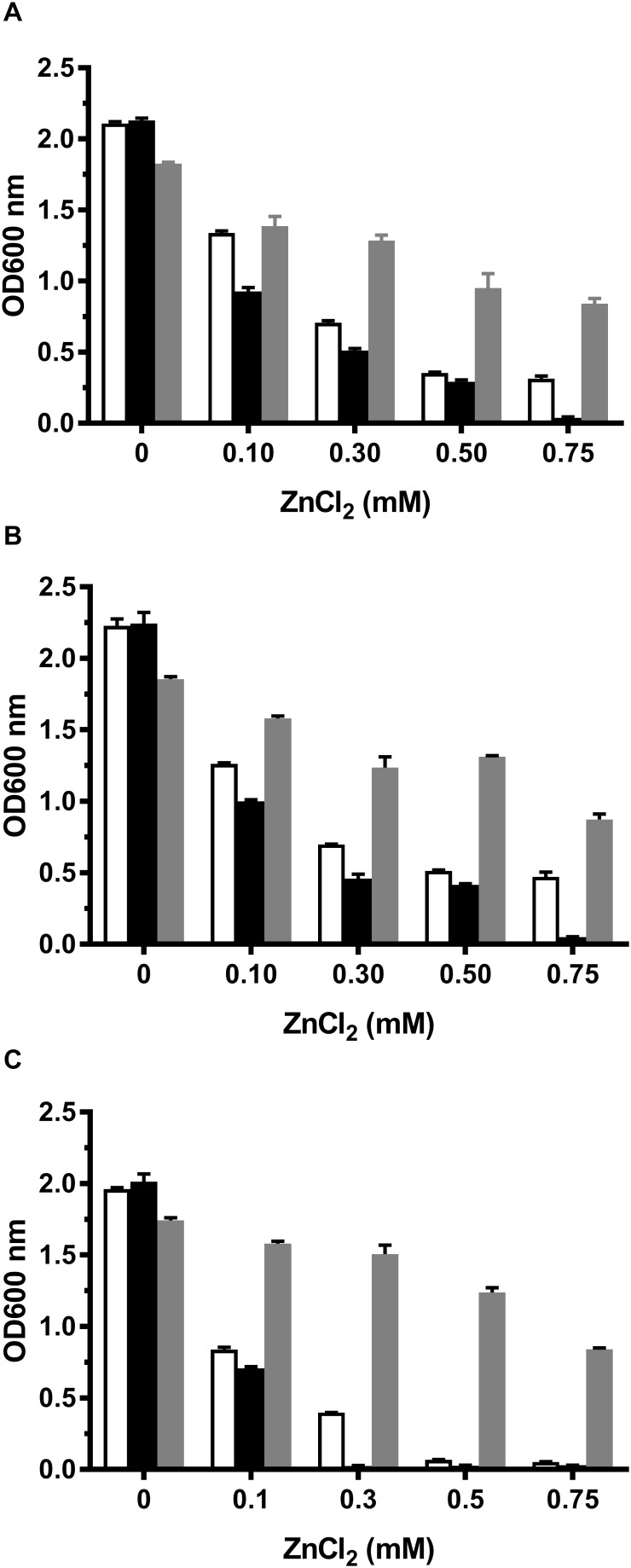
Growth tests for *E. coli* KZAB04 transformants in LB, LBK, and LBO broths containing different ZnCl_2_ concentrations. To test whether MceT functions as a Zn^2+^ efflux transporter, *E. coli* KZAB04/pTrcHisB (white column), KZAB04/pTrcHisB-mceT (black column) and KZAB04/pTrcHisB-czcD (gray column) as the positive control of a Zn^2+^ efflux transporter were grown in LB **(A)** or LBK **(B)** broths containing 0–0.75 mM ZnCl_2_. Also, to test the effect of the presence of Na^+^ or K^+^ on Zn^2+^ diffusion facilitated by MceT, the above-mentioned *E. coli* KZAB04 transformants were grown in LBO **(C)** broths containing 0–0.75 mM ZnCl_2_. The pre-cultures of *E. coli* KZAB04 transformant cells were grown to OD_600__nm_ at 1.0 in LBO broth at pH 7.0 at 37°C. The above-mentioned cell growth was ended after 24 h and the values for OD_600__nm_ then evaluated. Each data point represents Mean ± SD of three independent cultures.

**FIGURE 5:**
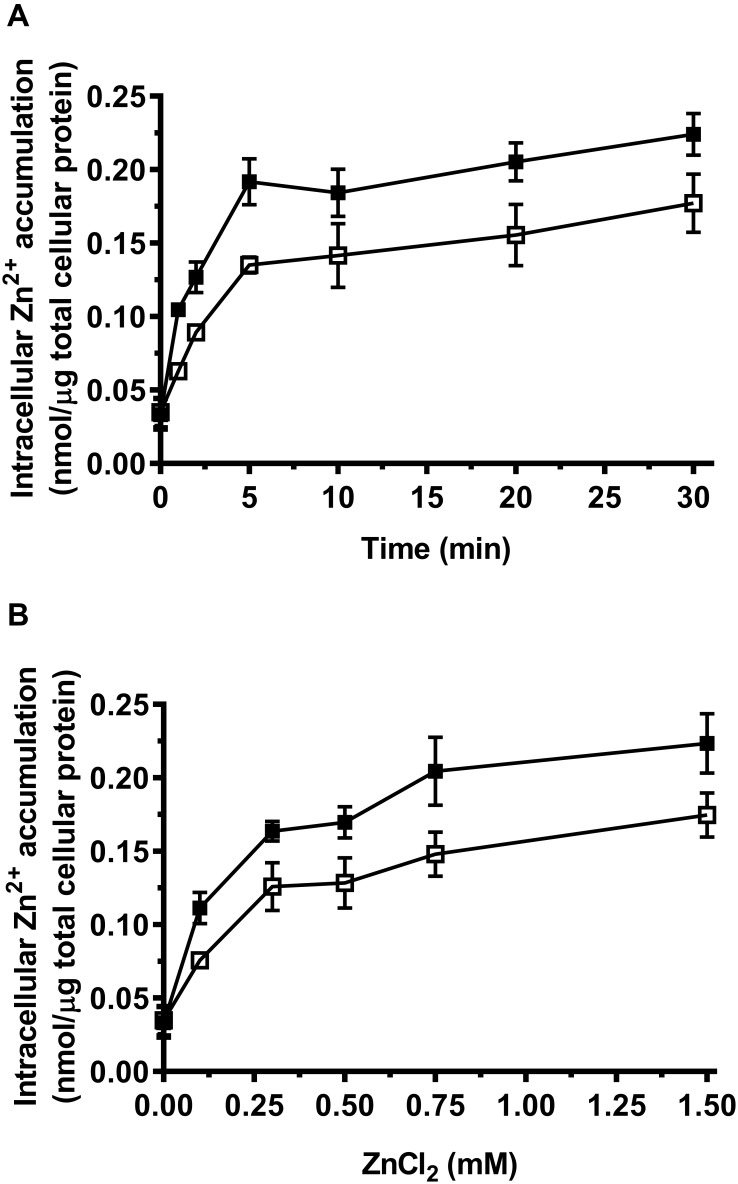
Comparison of intracellular Zn^2+^ accumulation between *E. coli* KZAB04/pTrcHisB-mceT and KZAB04/pTrcHisB. *| E. coli* KZAB04/pTrcHisB-mceT (filled square) and KZAB04/pTrcHisB as a negative control (open square) were grown to OD_600__nm_ at 1.0, respectively. Aliquots of prepared cells with the equal total protein were incubated at 0.75 mM ZnCl_2_ from 0 to 30 min **(A)** for the plotting of a time curve of intracellular accumulated Zn^2+^ contents and also incubated within 20 min at the different ZnCl_2_ concentrations ranging from 0 to 1.50 mM **(B)** for the comparison of intracellular accumulated Zn^2+^ contents between *E. coli* KZAB04/pTrcHisB-mceT and KZAB04/pTrcHisB. Each data point represents Mean ± SD of three independent experiments.

Among identified CDF members, MceT shares relatively higher cover range and identity with Zn-CDF members such as ZnT2 from *Rattus norvegicus*, ZnT4 from *R. norvegicus* and *Homo sapiens* (Supplementary Table 3 and Supplementary Figure 3). Therefore, Zn^2+^ was chosen as its potential substrate to test whether MceT can function as a Zn^2+^ efflux transporter. For this purpose, a Zn^2+^-sensitive *E*. *coli* mutant KZAB04 was constructed through the disruption of two major Zn^2+^ efflux transporters, ZntA (Beard et al., 1997; Rensing et al., 1997), and ZitB (Grass et al., 2001). Also, *B. subtilis* CzcD (Guffanti et al., 2002) was constructed as the positive control of a prokaryotic Zn^2+^ efflux transporter into an expression vector pTrcHisB (Supplementary Table 1). At first, growth tests were carried out in LB broths containing ZnCl_2_ concentrations varied from 0 to 0.75 mM (Figure 4A). Compared with *E. coli* KZAB04/pTrcHisB, the growth of *E. coli* KZAB04/pTrcHisB-czcD was significantly enhanced as ZnCl_2_ concentrations increased from 0.30 to 0.75 mM while that of KZAB04/pTrcHisB-mceT was slightly reduced the growth of *E. coli* in the presence of ZnCl_2_, even completely abolished at 0.75 mM ZnCl_2_ (Figure 5A). K^+^ was reported to be one of the coupling substrates of CzcD for exchange with the extracellular Zn^2+^ (Guffanti et al., 2002). Therefore, the growth of the above transformants was tested in LBK broths at the same tested ZnCl_2_ concentrations (Figure 4B). The growth trend in LBK broths was similar to that in LB broths, except that all the transformants showed higher growth in the former than in the latter. These results reveal that MceT can’t function as a Zn^2+^ efflux transporter, and also the growth failure of *E. coli* KZAB04/pTrcHisB-mceT at 0.75 mM ZnCl_2_ (Figure 4A,B) implies that Zn^2+^ sensitivity of *E. coli* KZAB04 should be exacerbated by MceT.

### Na^+^/K^+^ Independence of Zn^2+^ Sensitivity by MceT

To test whether the presence of Na^+^ or K^+^ may affect Zn^2+^ sensitivity by MceT, the growth tests were also performed in LBO broths with no addition of NaCl or KCl. In contrast to the empty vector pTrcHisB, expression of CzcD rendered the significant ZnCl_2_ resistance of *E. coli* KZAB04 in LBO broths (Figure 4C), which was similarly found in LB or LBK broths (Figure 4A,B). However, the major difference is that *E. coli* KZAB04 carrying pTrcHisB-mceT or pTrcHisB failed to grow in LBO broths containing 0.50 mM ZnCl_2_ and above (Figure 4C). That may be attributed to that the absence of Na^+^ or K^+^ inhibited the host growth, to some extent. Importantly, *E. coli* KZAB04/pTrcHisB could grow at 0.30 mM ZnCl_2_ while KZAB04/pTrcHisB-mceT lost the growth under the same stress (Figure 4C). These results not only confirm that Zn^2+^ sensitivity of *E. coli* KZAB04 was exactly exacerbated by MceT but also reveal that this phenomenon was not affected by the presence of Na^+^ or K^+^.

### Facilitated Diffusion of Zn^2+^ Into Cells by MceT

Considering Zn^2+^ sensitivity of *E. coli* KZAB04 was exacerbated by MceT, MceT may lose Zn^2+^ efflux activity but retain the Zn^2+^-binding ability and therefore facilitate the diffusion of Zn^2+^ into cells. To test this hypothesis, the difference in intracellular Zn^2+^ accumulation was compared between *E. coli* KZAB04/pTrcHisB-mceT and KZAB04/pTrcHisB under the stress of high ZnCl_2_ concentrations. As expected, *E*. *coli* KZAB04 cells expressing MceT accumulated significantly higher intracellular Zn^2+^ concentrations in the presence of 0.75 mM ZnCl_2_ than those with the empty vector, as the incubation time increased from 0 to 30 min (Figure 5A). Also, the similar difference was found between *E. coli* KZAB04/pTrcHisB-mceT and KZAB04/pTrcHisB within 20 min when ZnCl_2_ concentrations were varied from 0 to 1.50 mM (Figure 5B). These results reveal that expression of MceT indeed facilitate the diffusion of extracellular Zn^2+^ into the cells of *E. coli* KZAB04.

### A Representative of MceT as a Novel CDF Group

**FIGURE 6:**
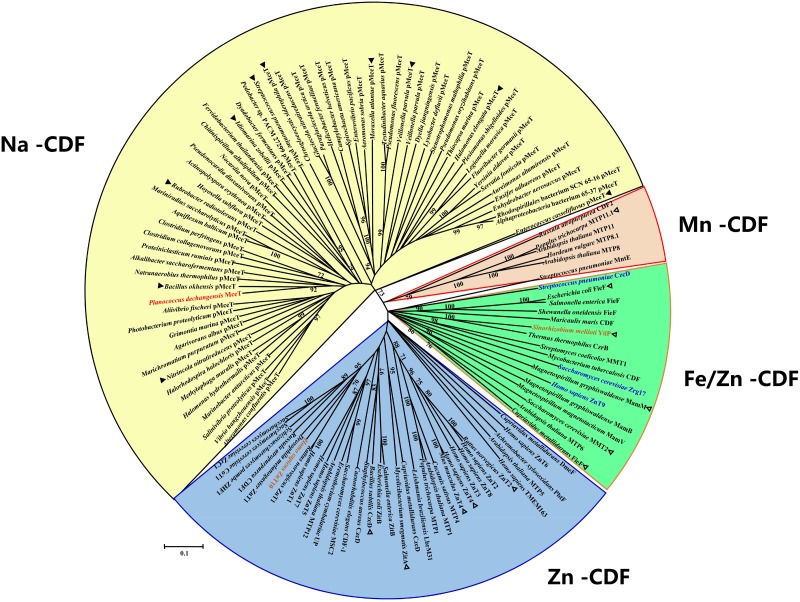
Neighbor-joining phylogenetic tree of MceT with CDF members. For the construction of phylogenetic tree, 62 putative MceT (pMceT) homologs were selected through BlastP at the NCBI website using the deduced amino acid sequence of MceT, and all the identified or putative CDF members collected in the TCDB database were also selected as the respective representatives of three known CDF groups including Zn-CDF (in a blue fan-shaped sector or three exceptional members in blue), Fe/Zn-CDF (in a green fan-shaped sector), and Mn-CDF (in a light red fan-shaped sector or one exceptional member in light red). MceT (in red) and its putative homologs are highlighted in a yellow fan-shaped sector. Ten homologs (filled triangle) were selected as the respective representatives of ten different clusters or clades and aligned with MceT, as shown in Supplementary Figure 2. Eleven identified CDF members (open triangle) with the query cover range above 50% were used for the alignment with MceT, as shown in Supplementary Figure 3. Accession version numbers of selected proteins were listed in the Supplementary Table 3. Bootstrap values ≥70% (based on 1000 replications) are shown at branch points. Bar, 0.1 substitutions per amino acid residue position.

On the basis of the above results, MceT may represent a novel class of CDF members. To establish this hypothesis, the phylogenetic relationship was analyzed using Neighbor-Joining (NJ) method between MceT and the representatives of identified or putative CDF members. For the accuracy and representativeness of the phylogenetic tree, the number of target sequences were set to the maximum numerical of 20,000 in the setting of BlastP algorithm parameters. Finally, 62 putative homologs with the identity range of 28–63% were selected and guaranteed to widely distribute in different species or strains from eight phyla as possible (Supplementary Table 3). All the identified or putative CDF members (Supplementary Table 3) were also downloaded from the TCDB database. To avoid biased group distribution, long gaps on extended N or C termini typical of some CDF members were extruded. As shown in Figure 6, CDF members from the TCDB database are clustered into three known CDF groups including Zn-CDF, Fe/Zn-CDF, and Mn-CDF, respectively, except for three exceptional Zn-CDF members such as *H. sapiens* ZnT9, *Saccharomyces cerevisiae* Zrg17 and *Streptococcus pneumoniae* CzcD and one exceptional Mn-CDF member, *Sinorhizobium meliloti* YiiP. This result reflects the reliability of the constructed phylogenetic tree. Interestingly, MceT exactly clustered with its 62 putative homologs with a bootstrap value of 73% (Figure 6). This suggests that MceT, together with its putative homologs, may constitute a novel CDF group designated Na-CDF with Na^+^ as the preferred substrate, which is significantly distant with three known CDF groups.

### Significantly Different Conserved Residues Located Within Motif 1 and Motif 3 of Na-CDF Group

**FIGURE 7:**
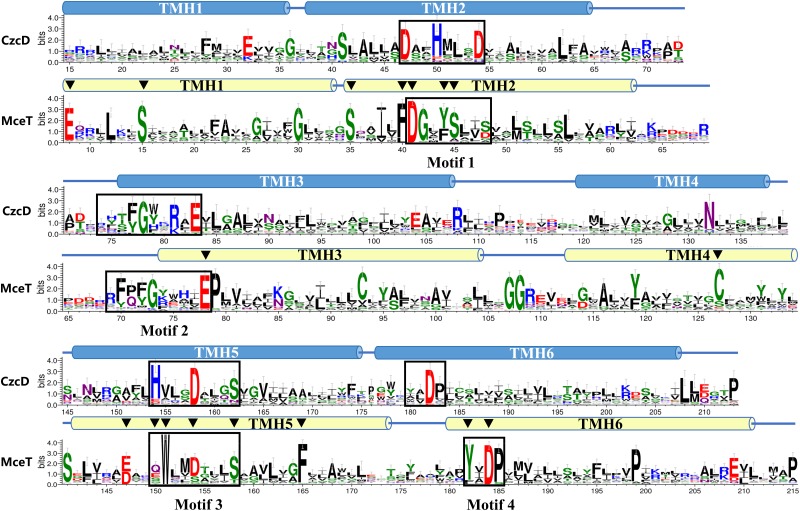
Comparison of web-based amino acid sequence logos between MceT homologs and identified CDF members. Amino acid sequence logos were created by submitting the alignment of MceT with its selected 62 homologs within the phylogenetic tree (Figure 6) and the alignment of *B. subtilis* CzcD with all the identified and putative CDF members downloaded from the TCDB database to the WebLogo 3 website http://weblogo.threeplusone.com/, respectively. The detailed information of proteins included in the phylogenetic tree is shown in Supplementary Table 3. The positions of residues correspond to those of CzcD and MceT, respectively. The heights of amino acid symbols stand for their conservation in the multiple alignment. Six predicted TMHs (TMH 1-6) of MceT (yellow filled cylinder) or CzcD (blue filled cylinder) were shown above the logos, respectively. Four conserved motifs within Na-CDF group and three known CDF groups are labeled and highlighted in the open rectangles. The highly conserved negatively-charged, polar or aromatic residues distributed within TMHs of MceT are marked with the black filled downward triangles above the logo of MceT and its homologs.

To establish whether MceT represents a novel CDF group, the web-based amino acid sequence logos were created to analyze the difference in conserved residues between Na-CDF group members and three known CDF groups. The polar or aromatic residues including Y44, S45, Q150, W151, and Y182 located within Motif 1 and Motif 3 were found to be highly conserved within Na-CDF group, which are different or non-conserved at the corresponding positions within three known CDF groups (Figure 7). Also, other negatively-charged, polar or aromatic residues such as E8, S15, F40, C93, C127, E147, and F165 unlocated within the four conserved motifs are highly conserved within Na-CDF group, which are not conserved at the corresponding positions within three known CDF groups (Figure 7). Moreover, negatively-charged or polar residues including S35 unlocated within the four conserved motifs, and D41, E78, D154, S158, and D184 located within the four conserved motifs are highly conserved between Na-CDF group and three known CDF groups. Exceptionally, S48 located within Motif 1 is not highly conserved between Na-CDF group members (Figure 7), although MceT shows significant difference at this position from eleven identified CDF members (Supplementary Figure 3). The most important finding is that “D41-Y44-S45” within Motif 1 of MceT and “Q150-D154” within Motif 3 of MceT are different from “D47-H50-D54” within Motif 1 of CzcD, and “H154-D158” within Motif 3 of CzcD, which supports that MceT, together with its putative homologs, should constitute a novel CDF group, Na-CDF.

### Functional Analysis of Conserved Residues Located Within TMHs of MceT

**FIGURE 8:**
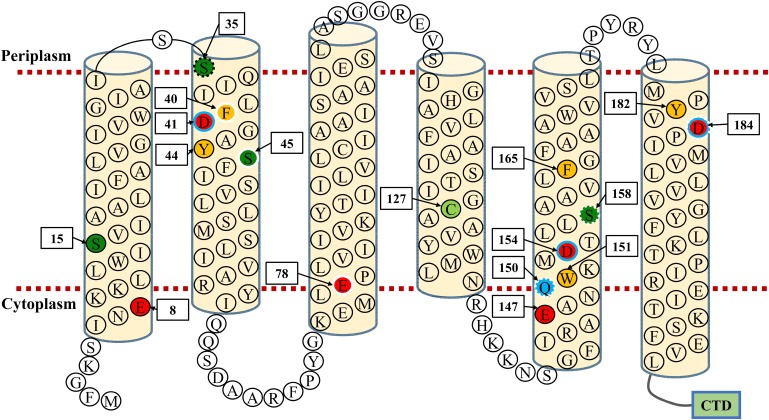
Topological model of MceT. This model is constructed based on the predicted topology using the deduced amino acid sequence of MceT. Six predicted TMHs (TMH 1–6) are marked with light yellow filled cylinders. Conserved negatively-charged residues (red filled circle), conserved aromatic residues (yellow filled circle), conserved serine residues (deep green filled circle), one conserved cysteine residue (light green filled circle) and one conserved glutamine residue (blue filled circle) are highlighted, respectively. S35, whose variant substituted by alanine showed no expression in the cytoplasmic membrane, is shown in the black dotted-line circle; three residues (F40, S45, E78), whose variants substituted by alanine significantly reduced the complementation capacity with *E. coli* KNabc, are shown in white solid-line circles; three residues (Q150, S158, D184), whose variants substituted by alanine lost the complementation capacity with *E. coli* KNabc, are shown in white dotted-line circles; three residues (D41, D154, D184), whose variants substituted by alanine rescued the growth of *E. coli* KZAB04, are shown in blue solid-line circles. CTD (green filled rectangle) stands for the C-terminal domain of MceT composed of 95 aa.

**FIGURE 9:**
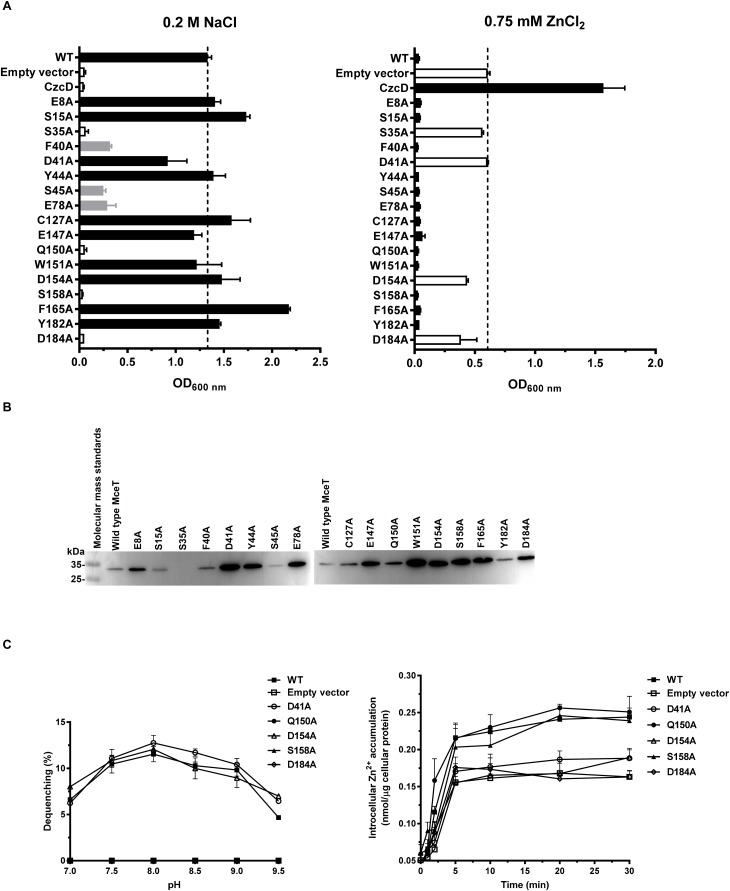
Functional analysis of MceT variants. To analyze the effect of MceT variants by site-directed mutagenesis on the Na^+^/H^+^ antiport activity or facilitated Zn^2+^ diffusion, *E. coli* KNabcor KZAB04 transformants **(A)** with the empty vector (negative control), and expressing CzcD (positive control as a Zn^2+^ efflux transporter), MceT or its variants substituted by alanine were grown in LBK broths containing 0.2 M NaCl or 0.75 mM ZnCl_2_ within 24 h, respectively, followed by the measurements of OD_600__nm_. To analyze the expression of MceT variants, western blot detection **(B)** was also carried out using everted membrane vesicles prepared from *E. coli* KNabc transformants of WT and its variants. The activity for Na^+^/H^+^ antiport and facilitated Zn^2+^ diffusion of MceT variants including Q150A, S158A, D41A, D154A, D184A was assayed **(C)**.

Conserved residues located within TMHs play a critical role in transporting activity of CDF transporters (Montanini et al., 2007; Hoch et al., 2012; Kolaj-Robin et al., 2015; Martin and Giedroc, 2016; Nishito et al., 2016) or Na^+^/H^+^ antiporters (Inoue et al., 1995; Habibian et al., 2005; Jiang et al., 2013). Based on the predicted topological model of MceT (Figure 8), 17 conserved residues excluding C93 and S48 were replaced to test their importance for Na^+^ efflux or facilitated Zn^2+^ diffusion. C127 was selected as a representative of C93 and C127, and also non-conserved S48 was not selected (Figure 7). Moreover, CzcD was chosen as the positive control of a Zn^2+^ efflux transporter and the empty vector as a negative control. For the functional analysis, *E. coli* KNabc and KZAB04 transformants expressing wild-type MceT or each of its variants were grown in LBK broth containing 0.2 M NaCl (Figure 9A, left panel) and LBK broth containing 0.75 mM ZnCl_2_ (Figure 9A, right panel), respectively. Expression of wild-type MceT and variants was verified in *E. coli* KNabc by western blot. In contrast to wild-type MceT, all its variants were identified to be expressed at the similar or higher level, with the sole exception of non-expressed S35A (Figure 9B). Two variants, Q150A and S158A, recovered no growth of *E. coli* KNabc in the presence of 0.2 M NaCl (Figure 9A, left panel), while still could inhibit the growth of *E. coli* KZAB04 in the presence of 0.75 mM ZnCl_2_ (Figure 9A, right panel). The activity assay showed that Q150A and S158A absolutely abolished Na^+^/H^+^ antiport activity (Figure 9C, left panel), indicating that Q150 and S158 are key residues of MceT only for Na^+^ efflux. Two variants, D41A and D154A, rendered Na^+^ tolerance of *E. coli* KNabc (Figure 9A, left panel) but completely or largely restored ZnCl_2_ resistance of *E. coli* KZAB04 (Figure 9A, right panel). Also, D41A and D154A lost the capability of facilitated Zn^2+^ diffusion (Figure 9C, right panel), indicating that D41 and D154 are involved in Zn^2+^ diffusion facilitated by MceT. Notably, D184A restored not only entire NaCl sensitivity of *E. coli* KNabc but also partial ZnCl_2_ resistance of *E. coli* KZAB04, implying D184 is the prerequisite for both Na^+^ and Zn^2+^ transport. This is supported by the results of activity assays (Figure 9C). Moreover, *E. coli* KNabc expressing F40A, S45A or E78A showed weak growth (Figure 9A, left panel). This reveals that these three residues are related, to some extent, to Na^+^ efflux by MceT. The non-expressed S35A (Figure 9B) had no effect on NaCl sensitivity of *E. coli* KNabc or ZnCl_2_ resistance of *E. coli* KZAB04 (Figure 9A). The possible reason can’t be speculated for non-expression of S35A. However, this finding confirms that the expression of wild-type MceT indeed can exacerbate ZnCl_2_ sensitivity of *E. coli* KZAB04 and even inhibit the host growth.

### Predicted Na^+^ and Zn^2+^ Transport Models for MceT

**FIGURE 10:**
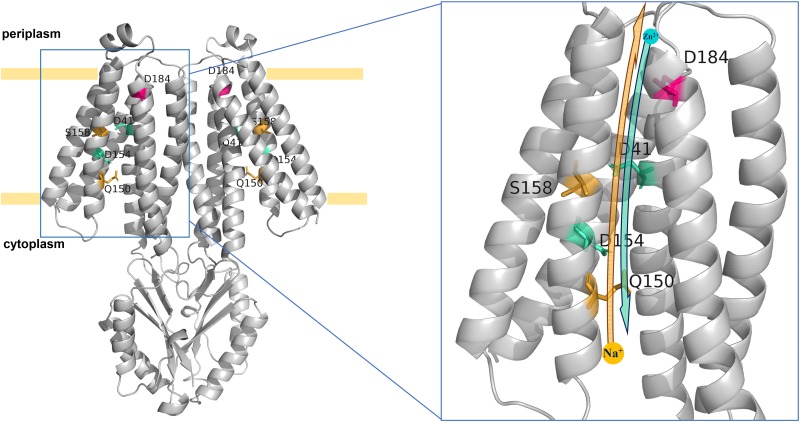
Predicted Na^+^ and Zn^2+^ transport models for MceT. The Na^+^ and Zn^2+^ transport models were predicted on the basis of the modeled structure of MceT with the template of 3D structure of *S. oneidensis* YiiP (PDB ID: 5vrf). Na^+^ and Zn^2+^ are drawn as a yellow filled circle and a cyan filled circle, respectively. Key residues including D41, Q150, D154, S158, and D184 are labeled and colored. The yellow and cyan arrows stand for the transport orientations of Na^+^ and Zn^2+^, respectively. In two models of MceT, MceT may employ the same ionic translocation channel for Na^+^ efflux and facilitated Zn^2+^ diffusion. In the presence of extracellular Na^+^, MceT can extrude Na^+^ in exchange for external H^+^ in a relay mode of three key Na^+^/H^+^-binding residues (Q150 > S158 > D184). Under the stress of high concentrations of extracellular Zn^2+^, MceT can facilitate the diffusion of Zn^2+^ into cells in a relay mode of three possible key Zn^2+^-transporting residues (D184 and D41 > D154).

In the predicted topological model of MceT (Figure 8), D41 in TMH2 and D184 in TMH6 are adjacent to the periplasmic side. Also, Q150, D154, and S158 are located in the center of TMH5, of which Q150 is closer to the cytoplasmic side and S158 closer to the periplasmic side. The modeled structure of MceT (Figure 10) with the template of 3D structure of *Shewanella oneidensis* YiiP (PDB ID: 5vrf) verifies the reliability of its predicted topological model and better shows the location of the above five key residues. Based on the current results, the Na^+^ and Zn^2+^ transport models for MceT were predicted, respectively (Figure 10). In these two models, MceT may employ the same transmembrane channel for Na^+^ and Zn^2+^ translocation (Figure 10). However, Q150, S158, and D184 are responsible for Na^+^ efflux and selectivity, and may transport Na^+^ outside the cells in a relay mode of Na^+^/H^+^ antiporters. In contrast, D41, D154 and D184 can still retain the capability of Zn^2+^ binding. D41 and D184 may form a metal ion coordinating site adjacent to the periplasmic side and thus can bind with the extracellular Zn^2+^. After their coordination of with Zn^2+^, D154 may act as a vital relay site and thus facilitate Zn^2+^ diffusion into cells under the stress of high concentrations of extracellular Zn^2+^.

## Discussion

This study identifies a novel CDF transporter, MceT, from the moderate halophile *P. dechangenesis* NEAU-ST10-9^T^. This transporter functions as a Na^+^(Li^+^, K^+^)/H^+^ antiporter, together with its facilitated Zn^2+^ diffusion into cells, which is significantly different from all identified CDF members (Paulsen and Saier, 1997; Haney et al., 2005; Montanini et al., 2007). MceT is proposed to represent a novel CDF group, Na-CDF, which shares relatively distant phylogenetic relationship with three known CDF groups including Mn-CDF, Fe/Zn-CDF, and Zn-CDF (Montanini et al., 2007). This can be strongly supported by a significant discrimination in key conserved residues of two function-related structural motifs between Na-CDF group and three known CDF groups. Site-directed mutagenesis implies that this discrimination leads to the evolution of MceT from a Zn^2+^-efflux transporter to a Na^+^(Li^+^, K^+^)/H^+^ antiporter. These presented findings provide the important evolutionary implications that CDF transporters can change the ionic selectivity from divalent cations to monovalent ones through the substitution of key conserved residues in their major structural motifs. More importantly, the discovery of MceT contributes to a typical transporter model of CDF family with the unique structural motifs, which will be utilized to explore the cation-selective mechanisms of secondary transporters.

In identified CDF transporters, four conserved coordinating residues in TMH2 and TMH5 are proposed as a major metal ion binding site (Coudray et al., 2013), in which the structural motifs such as DD-HD, HD-HD and ND-HD are responsible for the selection specificity between divalent cations including Zn^2+^, Cd^2+^, Fe^2+^, or Mn^2+^ (Montanini et al., 2007; Hoch et al., 2012; Kolaj-Robin et al., 2015; Martin and Giedroc, 2016; Nishito et al., 2016). At the corresponding positions of MceT, the residues in Motif 1 are varied to Y44 and S48 (YS) whereas the residues in Motif 3 are varied to Q150 and D154 (QD). This remarkable discrimination can elucidate the loss of Zn^2+^ efflux activity by MceT. Notably, YS-QD motifs in MceT remain a conserved aspartic acid residue (D154), which is consistent with the corresponding one of identified CDF members (Montanini et al., 2007; Hoch et al., 2012; Kolaj-Robin et al., 2015; Martin and Giedroc, 2016; Nishito et al., 2016). Mutation of D154A partially abolished the facilitated extracellular Zn^2+^ diffusion of MceT in cells, which suggests the Zn^2+^ coordinating ability of D154. The consistent conservation of D41 in Motif 1 and D184 in Motif 4 exists between Na-CDF and three known CDF groups. Mutations in either of two corresponding aspartic acid residues in two CDF members, *E. coli* ZitB and *Populus trichocarpa* ×*Populus deltoides* MTP1, completely abolished their resistance to Zn^2+^ (Blaudez et al., 2003; Anton et al., 2004; Montanini et al., 2007). Therefore, the corresponding residues of D41 and D184 may form an additional metal ion coordinating site in CDF transporters. In CDF family transporters, the metal ion coordinating ability of these two residues has not attracted enough attention. This may be because that their roles are masked by the existence of four coordinating residues in the shared structural motifs (Lu and Fu, 2007; Montanini et al., 2007; Lu et al., 2009; Hoch et al., 2012; Gupta et al., 2014; Kolaj-Robin et al., 2015; Martin and Giedroc, 2016; Nishito et al., 2016). However, varied motifs of MceT lack three key residues in the shared structural motifs of identified CDF transporters and thus highlight the significant roles of D41 and D184 in Zn^2+^ transport. Therefore, we propose to improve D-D (H, N)-D in TMH2, H-D in TMH5 and D in TMH6 as the common structural motifs of CDF members, which are responsible for the metal ion binding and/or selectivity.

It’s very interesting why and how MceT selects Na^+^ as the preferred substrate. 3D struture of *E. coli* NhaA provides a Na^+^-transporting model of Na^+^/H^+^ antiporters, in which D133, D163 and D164 constitute a core region for Na^+^ efflux in exchange with external H^+^ (Hunte et al., 2005). D184 is indispensable for both Na^+^ and Zn^2+^ transport of MceT, indicating that the aspartic acid residue at this position plays a decisive role in the cation coordination. Polar residues also play critical roles in the antiport activity of many Na^+^/H^+^ antiporters (Inoue et al., 1995; Habibian et al., 2005; Jiang et al., 2013). Mutations of Q150A and S158A led to the loss of Na^+^ tolerance, but had no effect on Zn^2+^ transport. The activity assay reveals that these two residues are closely involved in the Na^+^/H^+^ antiport activity of MceT, although it’s unclear whether they can bind with Na^+^ or H^+^. The negatively-charged aspartic acid and glutamic acid residues play predominant roles in the Na^+^ binding or protonation of Na^+^/H^+^ antiporters (Inoue et al., 1995; Habibian et al., 2005; Hunte et al., 2005; Jiang et al., 2013). However, a key glutamine residue sets Na^+^ pump rhodopsins from marine bacteria apart from H^+^ pump rhodopsins from marine bacteria, which may determine the Na^+^ coupling of Na^+^ pump rhodopsins (Inoue et al., 2015). Therefore, Q150 may act as a determinant for the selectivity of MceT for Na^+^.

Halophiles, which can grow at a wide range of 0.5–32.5% (w/v) NaCl, have to confront the challenges to the presence of molar concentrations of salt ions or high alkaline pH conditions and therefore may be driven to evolve their proteins for the adaptation to the extremely saline-alkaline habitats (Ventosa et al., 1998; Oren, 1999). Na^+^/H^+^ antiporters play a predominant role in maintaining intracellular pH and Na^+^ homeostasis (Padan and Landau, 2016). In the phylogenetic analysis, MceT and its homologs form an independent group, which is significantly distant with three known CDF groups. Combined with the functional characterization of MceT, this implies that MceT may have evolved from a Zn^2+^-efflux transporter to a Na^+^/H^+^ antiporter. MceT shares the homologies and similar structures with identified CDF members, but the function-related structural motifs are varied in key residues. Therefore, MceT is very likely to have been modified from its native CDF family function to a Na^+^/H^+^ antiporter in an evolutionary strategy of the substitution of key conserved residues in its function-related structural motifs. During the evolution, MceT selected YS-QD motifs in TMH1 and TMH3 and thus lost the Zn^2+^ efflux activity, and also employs Q150 for the selectivity of Na^+^ at this important cation-selective site. Also, it may retain D184 in TMH6 as the Na^+^-binding site and S158 in TMH3 as an assistant for the ionic transport, since they are conserved within CDF members (Paulsen and Saier, 1997; Haney et al., 2005; Montanini et al., 2007) and also can be utilized for the participation in the Na^+^/H^+^ antiport (Inoue et al., 1995; Habibian et al., 2005; Hunte et al., 2005). Moreover, MceT is not essential to modify D41 In TMH1 or D154 in TMH3, due to their no effect on the Na^+^/H^+^ antiport. This also explains why MceT can facilitate extracellular Zn^2+^ diffusion into cells. MceT retains D41, D154, and D184, and thus can still employ them to bind with Zn^2+^. However, without the aid of key residues for Zn^2+^ efflux, MceT is forced to facilitate extracellular Zn^2+^ diffusion into cells under the stress of high concentrations of extracellular Zn^2+^.

Although the Na^+^ and Zn^2+^ transport models were predicted for MceT, the exact roles of function-related residues remain to be clarified. In the future study, it needs to be confirmed whether D184 can bind with Na^+^, Zn^2+^, or H^+^ and whether D41 and D154 can bind with Zn^2+^. Also, it is very worthy of being explored whether Q150 or even S158 can bind directly with Na^+^ and thus cause MceT to select Q150 and S158 for the Na^+^/H^+^ antiport. Therefore, we have over-expressed and purified MceT and its variants for the isothermal titration calorimetry (ITC) test in order to analyze the cation-binding ability of the above-mentioned residues. To better answer the above questions, we also plan to discover its structure using purified MceT on the basis of X-ray crystallography. Furthermore, it needs to be confirmed whether variation in structural motifs of MceT leads to the loss of Zn^2+^ efflux activity. We have been modifying the residues of MceT through the site-directed mutagenesis based on the Zn^2+^-transporting model of CzcD and hope to reconstruct MceT as a Zn^2+^-efflux transporter, as the functions of identified CDF members.

## Data Availability

The datasets generated for this study can be found in GenBank, No. MH845411.

## Author Contributions

TX and JJ contributed to the study design, analyzed the data, and revised the manuscript. TX performed the construction of expression vector, zinc-sensitive *E*. *coli* mutant, and protein variants. SH performed the cloning of *mceT*. TX, HC, JL, LS, XZ, QZ, YW, and SG contributed to the cultures, preparation of everted membrane vesicles, and transporting activity determination. TX drafted the manuscript.

## Conflict of Interest Statement

The authors declare that the research was conducted in the absence of any commercial or financial relationships that could be construed as a potential conflict of interest.
