# Supplementary material for: Implications for Cation Selectivity and Evolution by a Novel Cation Diffusion Facilitator Family Member From the Moderate Halophile Planococcus dechangensis
Source: Front Microbiol. 2019 Mar 22;10:607. doi: 10.3389/fmicb.2019.00607 (PMC6440370; doi:10.3389/fmicb.2019.00607)
Supplement: Supplementary file 1 [file Data_Sheet_1.docx]

Supplementary Material

Implications for cation selectivity and evolution by a novel cation diffusion facilitator family member from the moderate halophile *Planococcusdechangensis*

Tong Xu, Huiwen Chen, Jincheng Li, Shan Hong, Li Shao, Xiutao Zheng, Qiao Zou, Yuting Wang, Sijia Guo and Juquan Jiang^*^

*** Correspondence:** Juquan Jiang: [jjqdainty@163.com](mailto:jjqdainty@163.com)

| Strains or plasmids | Relevant phenotype or genotype | The source |
| --- | --- | --- |
| Strains | | |
| *Planococcus dechangensis* NEAU-ST10-9^T^ | Type strain of *Planococcus dechangensis*, a moderate halophile | Isolated from saline and alkaline soils in Dechang Township, Zhaodong City, China |
| *Escherichia coli* KNabc | *nhaA*::Km^R^,*nhaB*::Em^R^, *chaA*::Cm^R^ | Donated by Professor Terry A. Krulwich |
| *Escherichia coli* DH5α | F^-^, φ80d*lacZ* ΔM15, Δ(*lacZYA-argF*)U169, *deoR*, *recA1*, *endA1*, *hsdR17*(*rK^-^*, *mK^+^*), *phoA*, *supE44*, *λ^-^*, *thi^-1^*, *yrA96*, *relA1* | Takara Biotechnology (Dalian) Co., Ltd., China |
| *Escherichia coli* KZAB04 | A Zn^2+^-sensitive *E. coli* DH5α mutant (*zntA*::Km^R^, *zitB*::Gen^R^), which was obtained by homologous recombination of pKD46 and pBBR1MCS-2 or pBBR1MCS-5 | This study |
| *Bacillus subtilis* subsp. *subtilis* strain 168 | A model-strain for *B. subtilis* genetics, *trpC2* gene of which was mutagenized by X-ray or UV light | China Center for Type Culture Collection |
| Plasmids | | |
| pUC18 | Cloning vector | Takara Biotechnology (Dalian) Co., Ltd., China |
| pUC-S5 | pUC18 carrying a 2.7 kb DNA fragment including *hyp*, *terR*/*acrR* and *mceT* | This study |
| pTrcHisB | Over-expression vector | Thermo Fisher Scientific Inc., China |
| pTrcHisB-mceT | pTrcHisB carrying *mceT* gene | This study |
| pTrcHisB-czcD | pTrcHisB carrying *czcD* gene from *B. subtilis* subsp. *subtilis* strain 168 | This study |
| pKD46 | Red recombinase expression plasmid with ampicillin resistance | Donated by Dr. W. Todd Lowther |
| pBBR1MCS-2 | Template plasmid for a kanamycin resistance gene | Donated by Professor Jose Enrique Ruiz-Sainz |
| pBBR1MCS-5 | Template plasmid for a gentamicin resistance gene | Donated by Professor Jose Enrique Ruiz-Sainz |

**Supplementary Table 1.** Strains and plasmids used in the study.

**Supplementary Table 2.** Sequence of primers used in this study.

| Primer Name | Primer sequence |
| --- | --- |
| MceT-F | GCCGGATCCGATGTTTGGAAAATCAATTAAC (*Bam*HI underlined) |
| MceT-R | CGCCAAGCTTATAAATGAGTTTTCTTTGCC (*Hin*dIII underlined) |
| CzcD-F | GCCGGATCCGATGGGTCACAATCATAATGA (*Bam*HI underlined) |
| CzcD-R | CGCCAAGCTTAATGGTGATGGTGGTCAC (*Hin*dIII underlined) |
| MceT-E8A-F | GAAAATCAATTAACG**C**GAAAAAATTA |
| MceT-E8A-R | **G**CGTTAATTGATTTTCCAAACATCGG |
| MceT-S15A-F | AAATTATTATGGATA**G**CGGTGATTGCTG |
| MceT-S15A-R | **C**TATCCATAATAATTTTTTCTCGTTAA |
| MceT-S35A-F | GGGATTGCCATCTCT**G**CTCAAATCATT |
| MceT-S35A-R | **C**AGAGATGGCAATCCCCCAAACAATGC |
| MceT-F40A-F | TCTCAAATCATTTTG**GC**CGACGGCGCAT |
| MceT-F40A-R | **GC**CAAAATGATTTGAGAAGAGATGGCAAT |
| MceT-D41A-F | CAAATCATTTTGTTCG**C**CGGCGCATATTC |
| MceT-D41A-R | **G**CGAACAAAATGATTTGAGAAGAGATG |
| MceT-Y44A-F | TTGTTCGACGGCGCA**GC**TTCTTTCATC |
| MceT-Y44A-R | **GC**TGCGCCGTCGAACAAAATGATTTG |
| MceT-S45A-F | TTCGACGGCGCATAT**G**CTTTCATCAGC |
| MceT-S45A-R | **C**ATATGCGCCGTCGAACAAAATGATTTG |
| MceT-E78A-F | GGAAAAGAAATGTTGG**C**GCCCTTGGTC |
| MceT-E78A-R | **G**CCAACATTTCTTTTCCGTATGGAAAGC |
| MceT-C127A-F | ATCAGCACGATTGGA**GC**CGCCGCTGTCTA |
| MceT-C127A-R | **GC**TCCAATCGTGCTGATGGCTGCGAATAC |
| MceT-E147A-F | GGTTTTATCCGCGCAG**C**AGCGAACCAGTG |
| MceT-E147A-R | **G**CTGCGCGGATAAAACCCGAATTCTTTTTG |
| MceT-Q150A-F | CGCGCAGAAGCGAAC**GC**GTGGAAAATGGA |
| MceT-Q150A-R | **GC**GTTCGCTTCTGCGCGGATAAAACCCGA |
| MceT-W151A-F | GCAGAAGCGAACCAG**GC**GAAAATGGATAC |
| MceT-W151A-R | **GC**CTGGTTCGCTTCTGCGCGGATAAAAC |
| MceT-D154A-F | AACCAGTGGAAAATGG**C**TACTTTGCTCAG |
| MceT-D154A-R | **G**CCATTTTCCACTGGTTCGCTTCTGCGCG |
| MceT-S158A-F | ATGGATACTTTGCTC**GC**CGCGGCCGTGTTG |
| MceT-S158A-R | **GC**GAGCAAAGTATCCATTTTCCACTGGTTC |
| MceT-F165A-F | GCCGTGTTGTTCGGA**GC**CGCCGCCGCCTG |
| MceT-F165A-R | **GC**TCCGAACAACACGGCCGCGCTGAGCAA |
| MceT-Y182A-F | AGATATTTGATGCCG**GC**TGTCGATCCGAT |
| MceT-Y182A-R | **GC**CGGCATCAAATATCTATAAGGCGTCA |
| MceT-D184A-F | TTGATGCCGTATGTCG**C**TCCGATAATGGT |
| MceT-D184A-R | **G**CGACATACGGCATCAAATATCTATAAGG |

The mutagenic nucleotides are shown in boldface.

**Supplementary Table 3.** 62 selected putative MceT homologs and CDF members collected in TCDB database.

| Phylum | Species or strain | Protein ID | Protein function | Accession version No.^a^ | Numbers of residues | Query covery (%)^b^ | Identity (%)^c^ |
| --- | --- | --- | --- | --- | --- | --- | --- |
| *Firmicutes* | *Planococcus dechangensis* | MceT | Na^+^(Li^+^, K^+^)/H^+^ antiporter and Zn^2+^ uptake transporter |  | 306 |  |  |
| Putative MceT homologs from NCBI website |  |  |  |  |  |  |  |
| *Actinobacteria* | *Actinopolyspora erythraea* | pMceT | putative CDF member, unknown function | WP_052428397.1 | 310 | 86 | 38 |
|  | *Hoyosella subflava* | pMceT | putative CDF member, unknown function | WP_013807274.1 | 303 | 95 | 44 |
|  | *Nocardia nova* | pMceT | putative CDF member, unknown function | WP_051494630.1 | 303 | 96 | 35 |
|  | *Pseudonocardia dioxanivorans* | pMceT | putative CDF member, unknown function | WP_013675858.1 | 305 | 97 | 40 |
|  | *Rubrobacter radiotolerans* | pMceT | putative CDF member, unknown function | WP_041338988.1 | 304 | 96 | 46 |
| *Bacteroidetes* | *Aquiflexum balticum* | pMceT | putative CDF member, unknown function | WP_084120697.1 | 303 | 97 | 45 |
|  | *Chitinispirillum alkaliphilum* | pMceT | putative CDF member, unknown function | KMQ51722.1 | 313 | 97 | 37 |
|  | *Dyadobacter fermentans* | pMceT | putative CDF member, unknown function | WP_015811644.1 | 305 | 97 | 33 |
|  | *Mariniradius saccharolyticus* | pMceT | putative CDF member, unknown function | WP_008623581.1 | 310 | 96 | 45 |
|  | *Pedobacter* sp. PACM 27299 | pMceT | putative CDF member, unknown function | WP_062547165.1 | 301 | 86 | 38 |
| *Cyanobacteria* | *Chroogloeocystis siderophila* | pMceT | putative CDF member, unknown function | WP_073550126.1 | 308 | 96 | 28 |
| *Deinococcus - Thermus* | *Deinococcus phoenicis* | pMceT | putative CDF member, unknown function | WP_034355767.1 | 306 | 96 | 31 |
| *Firmicutes* | *Alkalibacter saccharofermentans* | pMceT | putative CDF member, unknown function | WP_073270849.1 | 305 | 96 | 44 |
|  | *Bacillus okhensis* | pMceT | putative CDF member, unknown function | KHF41528.1 | 270 | 87 | 63 |
|  | *Clostridium collagenovorans* | pMceT | putative CDF member, unknown function | SHH73828.1 | 300 | 96 | 44 |
|  | *Clostridium perfringens* | pMceT | putative CDF member, unknown function | WP_003456050.1 | 268 | 86 | 42 |
|  | *Enterococcus casseliflavus* | pMceT | putative CDF member, unknown function | WP_005234104.1 | 306 | 95 | 33 |
|  | *Natranaerobius thermophilus* | pMceT | putative CDF member, unknown function | WP_012448550.1 | 299 | 95 | 43 |
|  | *Proteiniclasticum ruminis* | pMceT | putative CDF member, unknown function | WP_051651626.1 | 313 | 96 | 37 |
|  | *Streptococcus pneumoniae* | pMceT | putative CDF member, unknown function | COE63401.1 | 306 | 93 | 33 |
|  | *Veillonella parvula* | pMceT | putative CDF member, unknown function | WP_004695437.1 | 317 | 97 | 32 |
| *Proteobacteria* | *Aeromonas sobria* | pMceT | putative CDF member, unknown function | WP_042022364.1 | 308 | 96 | 33 |
|  | *Agarivorans albus* | pMceT | putative CDF member, unknown function | WP_016402759.1 | 220 | 68 | 36 |
|  | *Aliivibrio fischeri* | pMceT | putative CDF member, unknown function | WP_065641718.1 | 227 | 68 | 40 |
|  | *Alphaproteobacteria* bacterium 65-37 | pMceT | putative CDF member, unknown function | OJU46705.1 | 301 | 95 | 31 |
|  | *Alteromonas confluentis* | pMceT | putative CDF member, unknown function | WP_070123986.1 | 298 | 90 | 37 |
|  | *Aureimonas altamirensis* | pMceT | putative CDF member, unknown function | WP_060603261.1 | 302 | 96 | 30 |
|  | *Campylobacter helveticus* | pMceT | putative CDF member, unknown function | WP_082199220.1 | 317 | 97 | 30 |
|  | *Dyella jiangningensis* | pMceT | putative CDF member, unknown function | WP_038622124.1 | 300 | 97 | 28 |
|  | *Enhydrobacter aerosaccus* | pMceT | putative CDF member, unknown function | WP_085936989.1 | 302 | 95 | 29 |
|  | *Ensifer adhaerens* | pMceT | putative CDF member, unknown function | KDP73960.1 | 300 | 96 | 30 |
|  | *Enterovibrio pacificus* | pMceT | putative CDF member, unknown function | WP_068905469.1 | 304 | 95 | 31 |
|  | *Fluoribacter gormanii* | pMceT | putative CDF member, unknown function | WP_058466862.1 | 303 | 97 | 27 |
|  | *Glaciecola nitratireducens* | pMceT | putative CDF member, unknown function | WP_014110226.1 | 306 | 93 | 32 |
|  | *Grimontia marina* | pMceT | putative CDF member, unknown function | CZF86006.1 | 257 | 66 | 37 |
|  | *Halomonas elongata* | pMceT | putative CDF member, unknown function | WP_013332710.1 | 301 | 95 | 36 |
|  | *Halomonas hydrothermalis* | pMceT | putative CDF member, unknown function | WP_039175731.1 | 287 | 86 | 35 |
|  | *Halorhodospira halochloris* | pMceT | putative CDF member, unknown function | WP_025281679.1 | 303 | 81 | 42 |
|  | *Helicobacter fennelliae* | pMceT | putative CDF member, unknown function | WP_023947635.1 | 337 | 95 | 30 |
|  | *Idiomarina zobellii* | pMceT | putative CDF member, unknown function | WP_053953300.1 | 307 | 91 | 38 |
|  | *Legionella moravica* | pMceT | putative CDF member, unknown function | WP_028384233.1 | 304 | 96 | 28 |
|  | *Lysobacter defluvii* | pMceT | putative CDF member, unknown function | WP_052106578.1 | 320 | 96 | 32 |
|  | *Marichromatium purpuratum* | pMceT | putative CDF member, unknown function | WP_005221981.1 | 222 | 70 | 34 |
|  | *Marinobacter antarcticus* | pMceT | putative CDF member, unknown function | WP_072795041.1 | 289 | 83 | 40 |
|  | *Methylophaga muralis* | pMceT | putative CDF member, unknown function | WP_069296718.1 | 289 | 79 | 37 |
|  | *Moraxella atlantae* | pMceT | putative CDF member, unknown function | WP_067056731.1 | 358 | 95 | 30 |
|  | *Nitrincola nitratireducens* | pMceT | putative CDF member, unknown function | WP_051514496.1 | 304 | 82 | 38 |
|  | *Paraglaciecola arctica* | pMceT | putative CDF member, unknown function | WP_007619288.1 | 306 | 87 | 32 |
|  | *Photobacterium proteolyticum* | pMceT | putative CDF member, unknown function | WP_075763326.1 | 231 | 70 | 40 |
|  | *Plesiomonas shigelloides* | pMceT | putative CDF member, unknown function | SBT61161.1 | 302 | 95 | 28 |
|  | *Pseudomonas fluorescens* | pMceT | putative CDF member, unknown function | CAY46829.1 | 306 | 96 | 30 |
|  | *Pseudomonas oryzihabitans* | pMceT | putative CDF member, unknown function | WP_059315208.1 | 312 | 98 | 30 |
|  | *Rhodospirillales* bacterium SCN 65-16 | pMceT | putative CDF member, unknown function | ODT98266.1 | 301 | 95 | 31 |
|  | *Salinivibrio proteolyticus* | pMceT | putative CDF member, unknown function | WP_077675944.1 | 225 | 68 | 39 |
|  | *Serratia fonticola* | pMceT | putative CDF member, unknown function | WP_074028013.1 | 312 | 95 | 31 |
|  | *Stenotrophomonas maltophilia* | pMceT | putative CDF member, unknown function | WP_006373742.1 | 300 | 97 | 32 |
|  | *Testudinibacter aquarius* | pMceT | putative CDF member, unknown function | WP_048364416.1 | 301 | 96 | 29 |
|  | *Thiocapsa marina* | pMceT | putative CDF member, unknown function | WP_007192698.1 | 308 | 96 | 31 |
|  | *Vibrio hangzhouensis* | pMceT | putative CDF member, unknown function | WP_103878745.1 | 226 | 69 | 37 |
|  | *Yersinia aldovae* | pMceT | putative CDF member, unknown function | WP_004699457.1 | 312 | 95 | 33 |
| *Spirochaetes* | *Spirochaeta americana* | pMceT | putative CDF member, unknown function | WP_076487337.1 | 304 | 96 | 31 |
| *Thermotogae* | *Fervidobacterium thailandensis* | pMceT | putative CDF member, unknown function | WP_069293697.1 | 305 | 96 | 37 |
| Representative CDF members from TCDB database |  |  |  |  |  |  |  |
| *Actinobacteria* | *Streptomyces coelicolor* | MMT1 | putative CDF member, unknown function | CAB41061.1 | 234 | - | - |
|  | *Mycobacterium tuberculosis* | CDF | putative CDF member, unknown function | EFO73540.1 | 206 |  |  |
|  | *Mycobacterium smegmatis* | ZitA | Zn^2+^ efflux transporter | YP_885161.1 | 297 | 54 | 24 |
| *Chordata* | *Homo sapiens* | ZnT1 | Zn^2+^ efllux transporter | AAG53405.1 | 507 | - | - |
|  | *Homo sapiens* | ZnT2 | Zn^2+^ uptake transporter into endosomal / lysosomal vesicles | AAQ15245.1 | 323 | 46 | 27 |
|  | *Homo sapiens* | ZnT3 | Zn^2+^ uptake transporter into synaptic vesicles | NP_003450.2 | 388 | - | - |
|  | *Homo sapiens* | ZnT4 | zinc transporter | AAB82561.1 | 429 | 75 | 21 |
|  | *Homo sapiens* | ZnT5 | Zn^2+^ uptake transporter into Golgi or granules | NP_075053.2 | 765 | 28 | 24 |
|  | *Homo sapiens* | ZnT6 | Zn^2+^ uptake transporter into Golgi, endoplasmic reticulum, or granules | NP_060434.2 | 461 | - | - |
|  | *Homo sapiens* | ZnT7 | Zn^2+^ uptake transporter into Golgi or granules | NP_598003.2 | 376 | - | - |
|  | *Homo sapiens* | ZnT8 | Zn^2+^ transporter | AAM80562.1 | 369 | - | - |
|  | *Homo sapiens* | ZnT9 | Zn^2+^ transporter | NP_006336.3 | 568 | - | - |
|  | *Homo sapiens* | ZnT10 | Mn^2+^ transporter | AAP44332.1 | 485 | 11 | 29 |
|  | *Homo sapiens* | TMEM163 | Zn^2+^ transporter | AAH26170.1 | 289 | - | - |
|  | *Mus musculus* | ZnT4 | Zn^2+^ transporter | NP_035904.2 | 430 | 75 | 21 |
|  | *Rattus norvegicus* | ZnT1 | Zn^2+^ efflux transporter | NP_074044.1 | 507 | - | - |
|  | *Rattus norvegicus* | ZnT2 | Zn^2+^ uptake transporter into endosomal / lysosomal vesicles | AAB02775.1 | 359 | 67 | 23 |
| *Proteobacteria* | *Achromobacter xylosoxidans* | PbtF | Zn^2+^, Cd^2+^, Pb^2+^ efflux transporter | ADP19922.1 | 211 | - | - |
|  | *Cupriavidus metallidurans* | FieF | Fe^2+^, Zn^2+^, Cd^2+^, Co^2+^, Ni^2+^ efflux transporter | ABF10278.1 | 337 | 82 | 18 |
|  | *Cupriavidus metallidurans* | DmeF | Zn^2+^, Cd^2+^, Co^2+^ efflux transporter | ABF07084.1 | 382 | - | - |
|  | *Cupriavidus metallidurans* | CzcD | Zn^2+^, Cd^2+^, Co^2+^ efflux transporter | YP_145596.1 | 316 | - | - |
|  | *Escherichia coli* | FieF | Zn^2+^/Cd^2+^/Hg^2+^/Fe^2+^:H^+^ antiporter | NP_418350.1 | 300 | 56 | 20 |
|  | *Escherichia coli* | ZitB | Zn^2+^/Cd^2+^:H^+^ antiporter | NP_415273.1 | 313 | - | - |
|  | *Salmonella enterica* | FieF | Zn^2+^/Cd^2+^/Hg^2+^/Fe^2+^:H^+^ antiporter | Q8ZKR4.1 | 300 | 40 | 22 |
|  | *Salmonella enterica* | ZitB | Zn^2+^/Cd^2+^:H^+^ antiporter | Q8ZQT3.1 | 312 | - | - |
|  | *Magnetospirillum gryphiswaldense* | MamB | heterodimeric stable complex with MamM as a putative magnetosome membrane Fe^2+^ transporter | CAE12043.1 | 297 | - | - |
|  | *Magnetospirillum gryphiswaldense* | MamM | heterodimeric stable complex with MamB as a putative magnetosome membrane Fe^2+^ transporter | CAJ30120.1 | 318 | 61 | 24 |
|  | *Magnetospirillum magnetotacticum* | MamV | putative Zn^2+^, Cd^2+^, Co^2+^ efflux transporter, but unknown function | KIM00465.1 | 322 | 49 | 25 |
|  | *Maricaulis maris* | CDF | Zn^2+^, Cd^2+^ efflux transporter | ABI64412.1 | 323 | - | - |
|  | *Shewanella oneidensis* | FieF | Zn^2+^, Cd^2+^ efflux transporter | AAN57440.1 | 296 | - | - |
|  | *Sinorhizobium meliloti* | YiiP | Mn^2+^ efflux transporter | CAC46965.1 | 306 | 58 | 22 |
| *Arthropoda* | *Drosophila melanogaster* | ZnT1 | Zn^2+^ efflux transporter | AGB94059.1 | 545 | - | - |
| *Ascomycota* | *Eremothecium cymbalariae* | UP | putative CDF member, unknown function | AET39232.1 | 701 | - | - |
|  | *Saccharomyces cerevisiae* | MSC2 | nuclear / endoplasmic reticulum Zn^2+^ uptake transporter | NP_010491.2 | 724 | 26 | 21 |
|  | *Saccharomyces cerevisiae* | CoT1 | Zn^2+^, Cd^2+^, Co^2+^ efflux transporter | DAA11080.1 | 439 | 29 | 29 |
|  | *Saccharomyces cerevisiae* | ZrC1 | Zn^2+^, Cd^2+^, Co^2+^ efflux transporter | NP_013970.1 | 442 | 32 | 25 |
|  | *Saccharomyces cerevisiae* | Zrg17 | Nuclear / endoplasmic reticulum Zn^2+^ uptake transporter | NP_014437.1 | 605 | - | - |
|  | *Saccharomyces cerevisiae* | MMT2 | mitochondrial Fe^2+^ transporter | CAA97939.1 | 484 | 75 | 22 |
|  | *Schizosaccharomyces pombe* | ZHF1 | Zn^2+^, Cd^2+^, Co^2+^ efflux transporter | O13918.3 | 387 | - | - |
| *Basidiomycota* | *Russula atropurpurea* | CDF1 | vacuolar Zn^2+^, Cd^2+^, Co^2+^ transporter, but not Mn^2+^ | AMM70772.1 | 487 | 23 | 27 |
|  | *Russula atropurpurea* | CDF2 | Zn^2+^, Co^2+^ transporter, but not Cd^2+^, Mn^2+^ | AMM70773.1 | 417 | - | - |
| *Deinococcus-Thermus* | *Thermus thermophilus* | CzrB | Zn^2+^, Co^2+^ transporter | CAC83722.1 | 291 | - | - |
| *Firmicutes* | *Bacillus subtilis* | CzcD | Zn^2+^, Cd^2+^, Co^2+^ efflux transporter | NP_390542.1 | 311 | 70 | 22 |
|  | *Staphylococcus aureus* | ZntA | Zn^2+^, Co^2+^ efflux transporter | AAC32485.1 | 326 | 31 | 21 |
|  | *Streptococcus pneumoniae* | CzcD | Zn^2+^ efflux transporter | AAL00475.1 | 299 | 8 | 33 |
|  | *Streptococcus pneumoniae* | MntE | Mn^2+^ efflux transporter | AAK75639.1 | 320 | - | - |
| *Kinetoplastida* | *Leishmania braziliensis* | LbrM31 | Zn^2+^ transporter | XP_001567260.1 | 443 | - | - |
| *Nematoda* | *Caenorhabditis elegans* | CDF-1 | Zn^2+^ efflux transporter | NP_509096.1 | 561 | 49 | 25 |
| *Streptophyta* | *Arabidopsis thaliana* | MTP1 | Zn^2+^ transporter | NP_182203.1 | 398 | - | - |
|  | *Cucumis sativus* | MTP4 | Zn^2+^, Cd^2+^ efflux transporter | AFJ24701.1 | 386 | - | - |
|  | *Arabidopsis thaliana* | MTP5 | heteromeric complex with MTP12 as a Zn^2+^ uptake transporter into Golgi | AEE75149.1 | 393 | - | - |
|  | *Arabidopsis thaliana* | MTP6 | putative Fe^2+^, Zn^2+^ transporter, but unknown function | AEC10893.1 | 471 | 90 | 23 |
|  | *Arabidopsis thaliana* | MTP8 | Mn^2+^ transporter | AEE79737.1 | 411 | 32 | 23 |
|  | *Arabidopsis thaliana* | MTP11 | trans-Golgi localized Mn^2+^ transporter | AEC09679.1 | 298 | 16 | 23 |
|  | *Arabidopsis thaliana* | MTP12 | heteromeric complex with MTP5 as a Zn^2+^ uptake transporter into Golgi | AAD32753.1 | 300 | - | - |
|  | *Hordeum vulgare* | MTP8.1 | tonoplast-localized Mn^2+^ transporter | AFP33387.1 | 400 | 20 | 26 |
|  | *Populus trichocarpa* | MTP1 | vacuolar Zn^2+^ transporter | AAR23528.1 | 393 | 33 | 31 |
|  | *Populus trichocarpa* | MTP11.1 | Golgi / endomembrane Mn^2+^-specific transporter | XP_002315247.1 | 394 | 64 | 18 |

The deduced amino acid sequence of MceT was aligned by using BlastP at the NCBI website and then its homologs were downloaded. All representative CDF members were downloaded from the TCDB database. ^a^ accession version number of Genbank database; ^b^ cover range of the number of amino acid residues of the aligned proteins to that of MceT; ^c^ identity of the aligned proteins with MceT within the range of covered residues; "-" means that MceT shares no query cover range or identity with the aligned proteins.


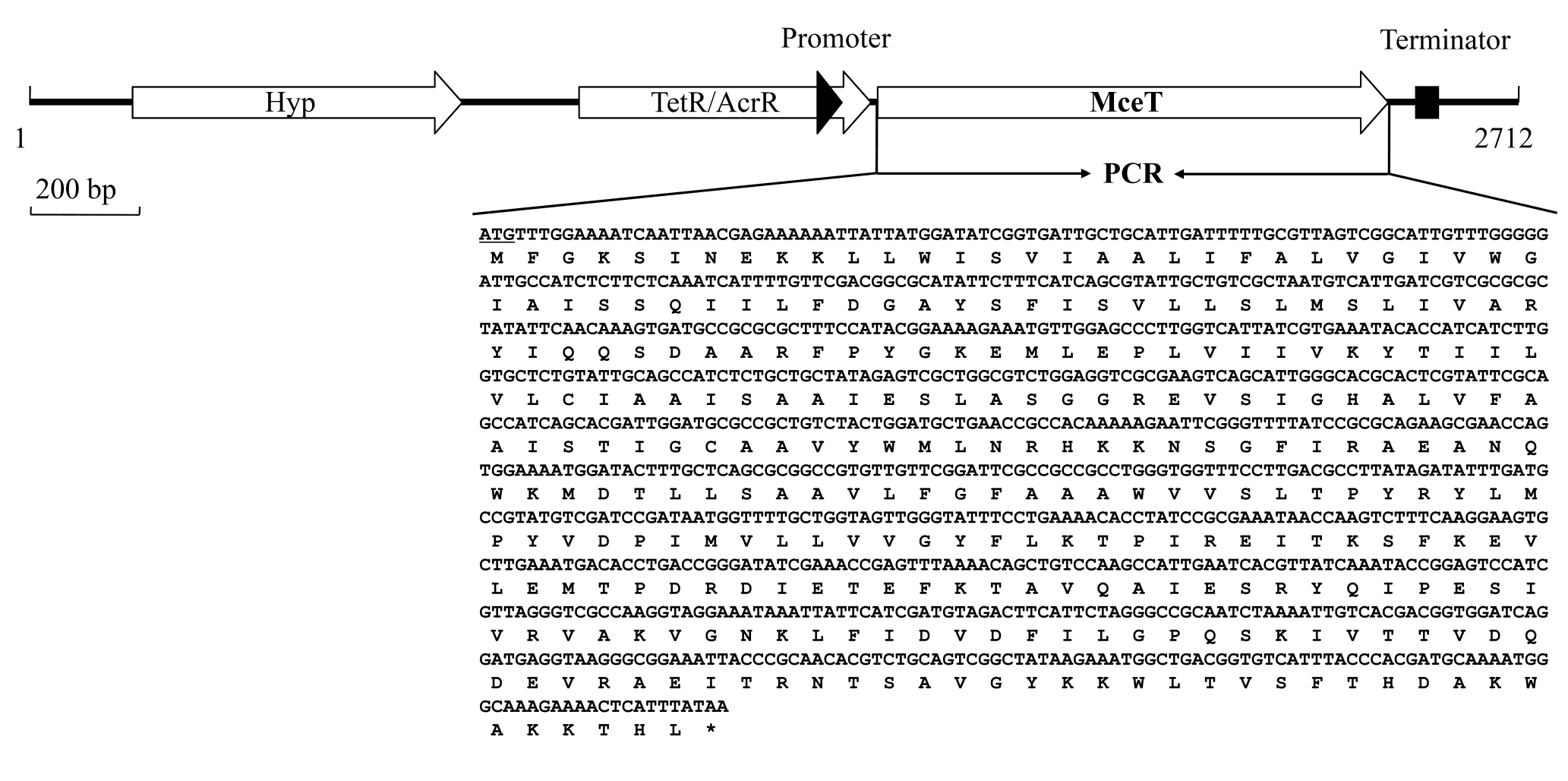


**Supplementary Figure 1.** Mapping of the inserted DNA fragment in the recombinant plasmid pUC-S5 and subcloning of *mceT* gene.

One hypothetical protein (Hyp), one predicted TetR/AcrR family regulator and MceT are included in this 2.7-kb DNA fragment. *mceT* is preceded by respective promoter-like sequence (filled arrow) and followed by a possible terminator (filled rectangle). The sequence of *mceT* gene from its initiation codon (ATG, underlined) to stop codon (TAA, marked with the asterisk) was subcloned by PCR amplification and fused in frame with an N-terminal 6×His tag into an expression vector pTrcHisB.


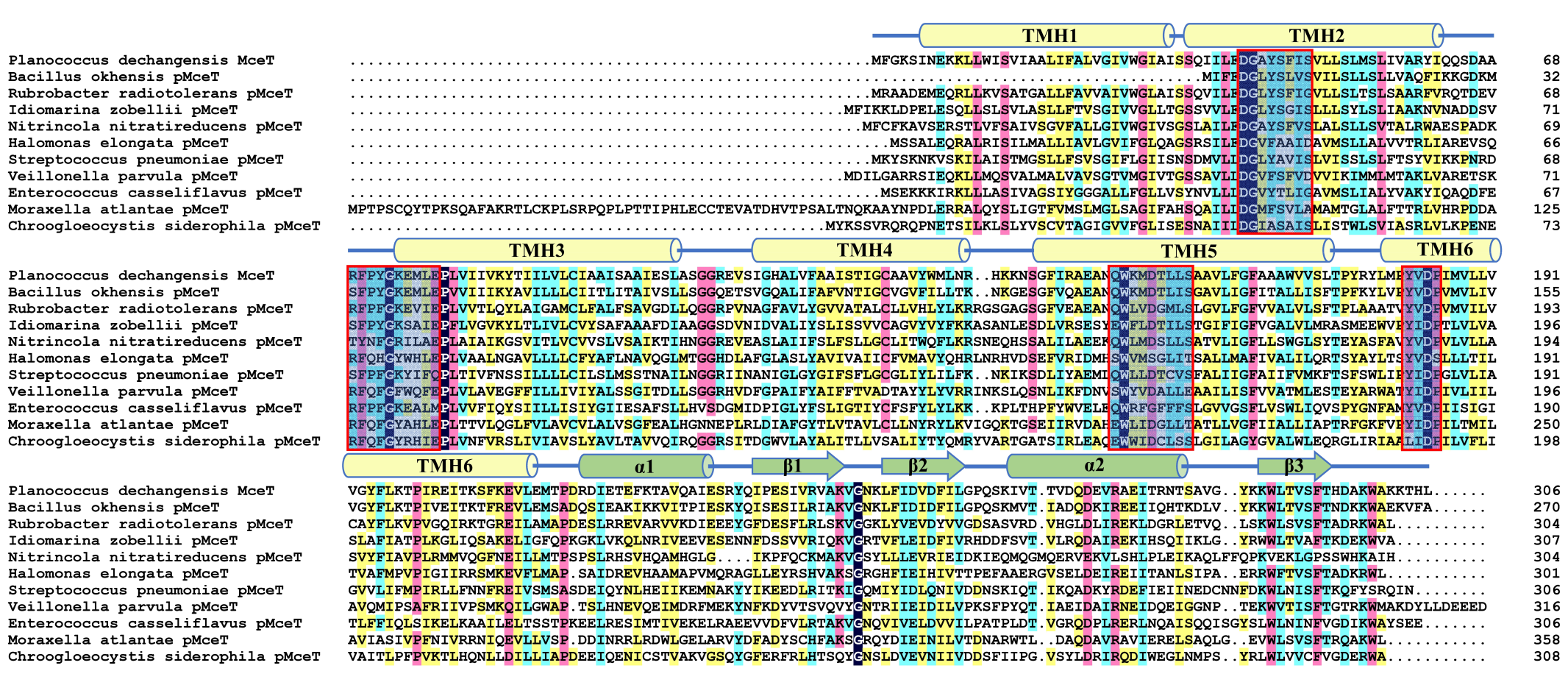


**Supplementary Figure 2.** Alignment of MceT with its ten selected homologs.

Ten homologs were selected as the respective representatives of ten different clusters or clades and aligned with MceT. Accession version numbers of selected proteins were listed in the Supplementary Table 3. Shading homology corresponds to 100% (black), ≥ 75 % (pink), ≥ 50% (cyan), and ≥ 33 % (yellow) amino acid identity, respectively. Six predicted transmembrane helices (light yellow filled cylinder), two α cytoplasmic helixes (light green filled cylinder) and three β strands (light green filled arrow) are shown above the alignment. Four conserved motifs are highlighted in light blue frames with red borders.


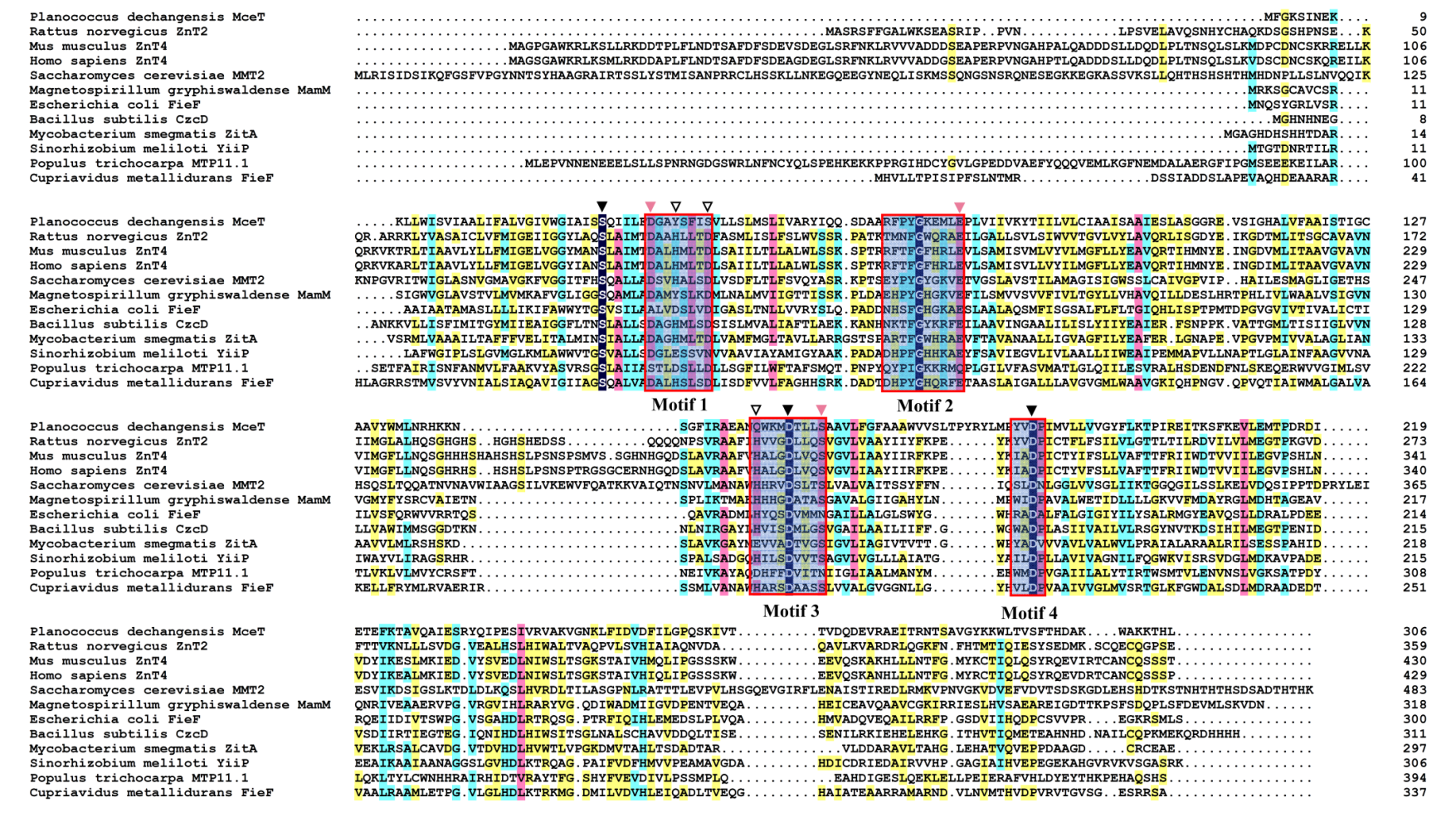


**Supplementary Figure 3.** Alignment of MceT with eleven identified CDF members.

MceT was aligned by using BlastP at the NCBI website with all CDF members collected in TCDB database. Eleven identified CDF members with query cover range above 50 % (Supplementary Table 3) were selected to show the alignment with MceT. Shading homology corresponds to 100% (black), ≥ 75 % (pink), ≥ 50% (cyan), and ≥ 33 % (yellow) amino acid identity, respectively. Four conserved motifs are highlighted in light blue frames with red borders. Fully conserved polar or negatively-charged (black filled downward triangle), highly conserved polar or negatively-charged (pink filled downward triangle) and significantly different (open downward triangle) amino acid residues located within the above-mentioned four motifs are shown above the alignment.


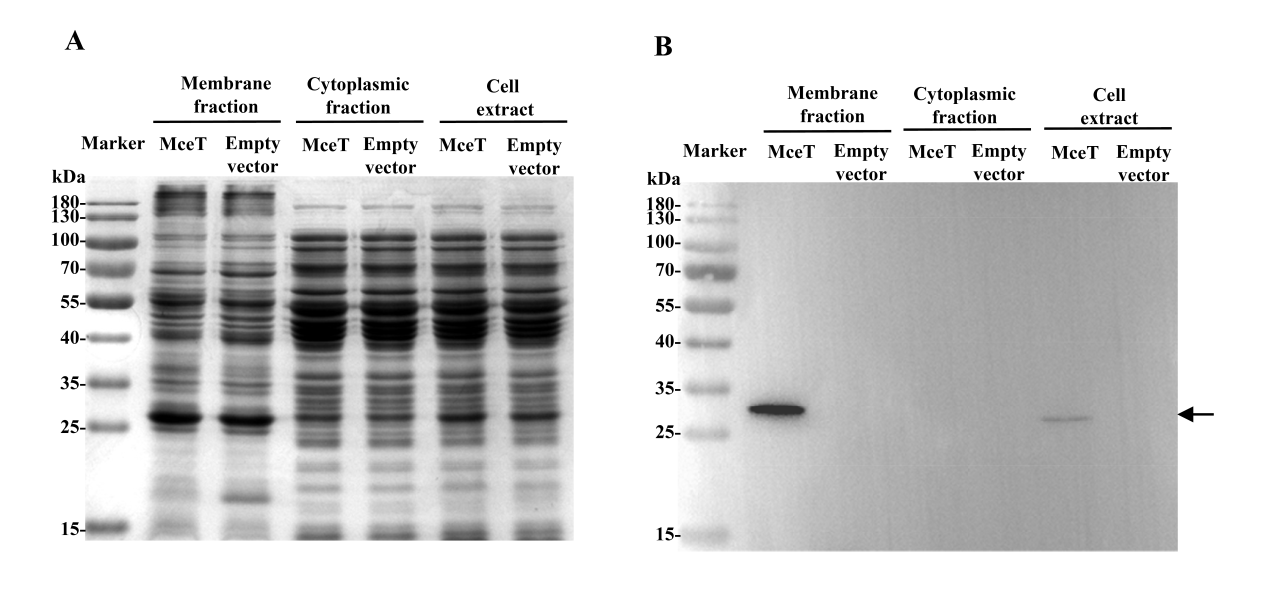


**Supplementary Figure 4.** Localization of MceT by western blot in the cytoplasmic membranes of *E. coli* KNabc.

For the establishment of MceT being a transmembrane protein, the samples for membrane fraction, cytoplasmic fraction and cell extract were prepared from cells of *E. coli* KNabc with pTrcHisB-mceT or the empty vector pTrcHisB as a negative control, followed by the analyses of SDS-PAGE (A) and western blot (B). The position of the target protein MceT fused with an N-terminal 6×His tag shown with a solid arrow.
